# Fluorous-Directed Clamping Stabilizes Triple-Helical DNA

**DOI:** 10.1021/acsomega.6c00803

**Published:** 2026-06-01

**Authors:** Andrea Taladriz-Sender, Michael Brazzill, Jamie M. Withers, Alasdair W. Clark, Glenn A. Burley, David A. Rusling

**Affiliations:** † Department of Pure Applied Chemistry, 3527 University of Strathclyde, Thomas Graham Building, 295 Cathedral Street, Glasgow G1 1XL, U.K.; ‡ School of Medicine, Pharmacy and Biomedical Sciences, 6697 University of Portsmouth, Portsmouth PO1 2DT, U.K.; § James Watt School of Engineering, Advanced Research Centre, 3526 University of Glasgow, Glasgow G11 6EW, U.K.; ∥ Strathclyde Centre for Molecular Bioscience, University of Strathclyde, Glasgow G1 1XQ, U.K.

## Abstract

Here we present a strategy to stabilize DNA triplexes by the attachment of perfluorinated tails to the 3′ and/or 5′ end of oligodeoxyribonucleotides. The tails preferentially associate with one another via the “fluorous effect” without compromising sequence selectivity. The most stable complex resulted in a dramatic 14 °C increase in melting temperature at neutral pH. This orthogonal recognition approach broadens the applications of triplex DNA in diagnostics, therapy, and DNA nanotechnology.

## Graphical abstract

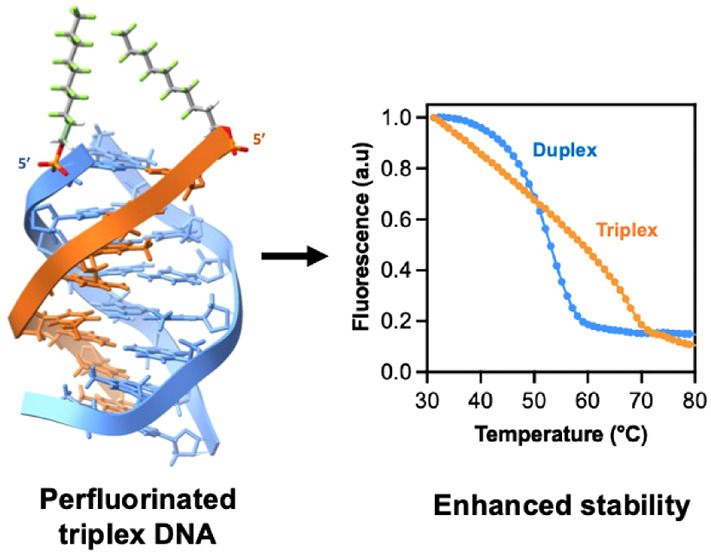

## Introduction

Triplex-forming oligonucleotides (TFOs) are sequence-selective DNA recognition agents that can be programmed to bind to unique double-stranded (DS) sequences by Hoogsteen hydrogen bonding within the duplex major groove (e.g., Figure 1A).^1,2^ Additionally, TFO clamps can be designed to bind to single-stranded (SS) nucleic acids by forming both Watson–Crick and Hoogsteen hydrogen bonds, generating more stable triplex complexes.^3–6^ Triplex structures offer applications in diagnostics, where they can be used for the targeted detection of specific nucleic acid sequences; in therapy, particularly in the modulation of transcription; and in nanotechnology, where they contribute to the design and assembly of complex nucleic acid architectures.^7–10^

**Figure 1:**
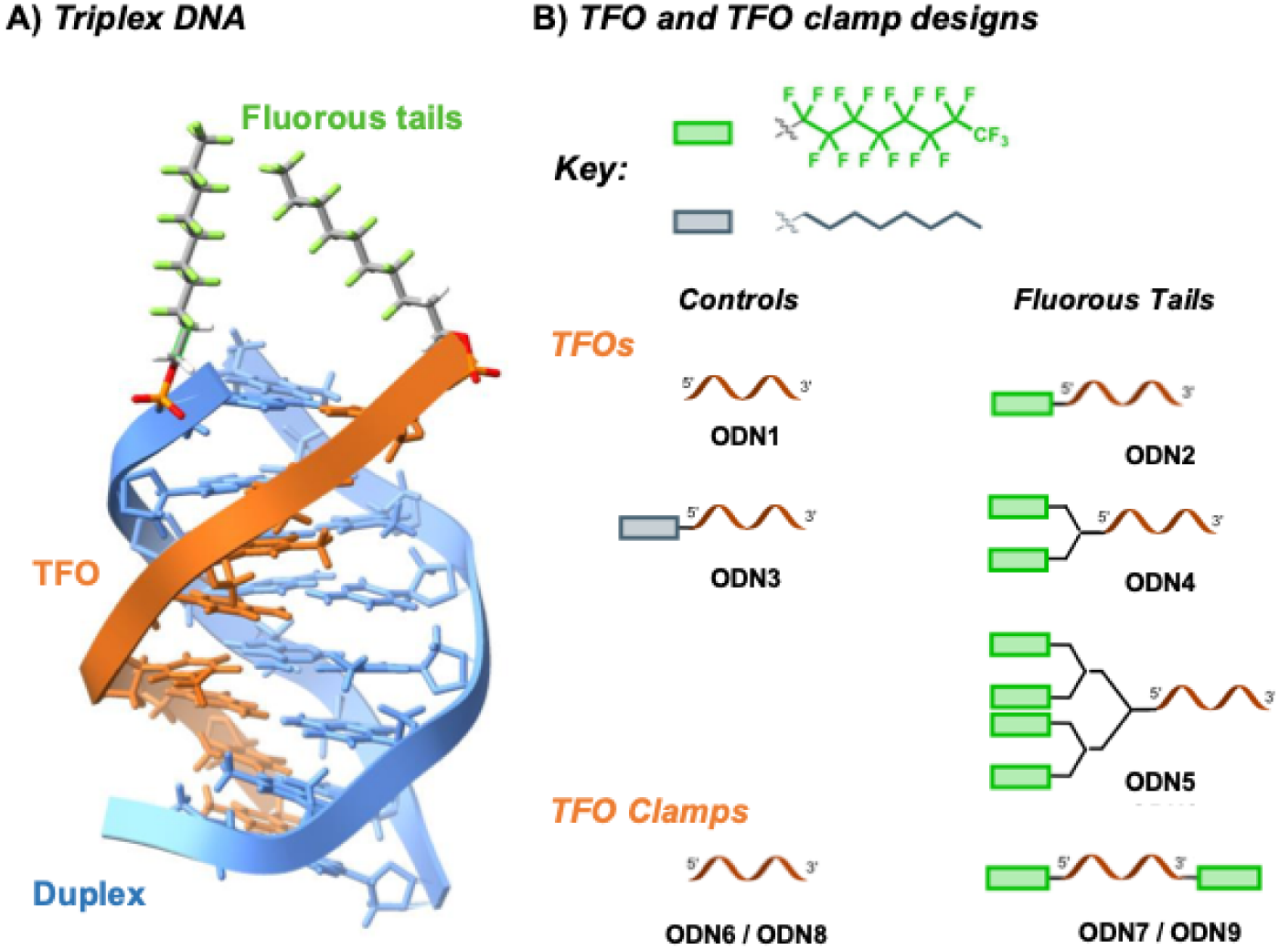
Triplex clamping directed by the fluorous effect. (A) Model of a fluorous-modified triplex based on an NMR structure of a parallel triplex containing C^+^:G-C and T:A-T triplets (PDB code: 1D3X). (B) Schematic illustration of the triplex-forming oligonucleotides investigated in this study. TFO and TFO clamps (ODN) are shown in orange (ODN1–9), the duplex is shown in blue, perfluorinated tails (RF) are shown in green (RF, RF2, and RF4 corresponding to 1, 2, and 4 tails, respectively), and the alkyl tail used as a control is shown in gray.

Binding of the third strand is dictated by the base composition of the oligonucleotide, with pyrimidine-rich TFOs binding parallel to the purine strand of the target duplex, forming T:A-T and C^+^:G-C triplets. [Here, X:R-Y denotes a triplet, where the third strand base X interacts with the duplex base pair R-Y by Hoogsteen hydrogen bonding to base R.] However, the stability of triplexes composed of these triplets is inherently low because of a requirement for cytosine protonation in the third strand. Triplex stability can be increased by introducing base, sugar, and/or backbone modifications within the oligonucleotides.^7,11^ These modifications generally improve stability by increasing the number of Hoogsteen hydrogen bonds, enhancing base stacking interactions, introducing positive charges, or altering the helicity of the triplex, with reports suggesting A-like helices are more stable.^12–14^ A drawback is that such modifications can reduce the sequence selectivity of oligonucleotide binding.^15^ An alternative strategy is to stabilize the structure through tethering groups to the ends of oligonucleotides that interact with nearby sequences.^16^ Such groups include positively charged polycations,^17^ intercalating agents,^18–20^ minor-groove binders,^21,22^ and cross-linking agents.^23^ Various other modalities, such as dendrimers,^24^ have also been used to stabilize nucleic acid complexes by their attachment to the underlying DNA. Orthogonal modes of molecular recognition distinct from W–C hybridization can either enhance or hinder the affinity and sequence selectivity of the oligonucleotides.^25,26^

Recently, the incorporation of perfluorinated tails within or at the termini of oligodeoxyribonucleotides (ODNs) has been shown to impart novel physicochemical properties on both oligonucleotides and their complexes.^27,28^ Perfluorinated groups preferentially associate with one another while excluding other hydrophilic and hydrophobic interactions, a phenomenon known as the “fluorous effect”.^29,30^ The attachment of fluorous tails to ODNs has been harnessed for a variety of applications, ranging from aiding purification,^31,32^ nanopatterning on surfaces,^33^ and higher-order DNA origami assembly.^34^ Fluorous-tagged ODNs also demonstrate enhanced cellular uptake and resistance to degradation by cellular nucleases,^35–37^ facilitating applications in cellular imaging^38^ and gene silencing.^39^ Given these unique characteristics, we therefore explored the triplex-forming properties of ODNs end-modified with perfluorinated groups and assessed the stability of the resultant complexes (Figure 1B).

## Results and Discussion

In our previous work, we investigated how perfluorinated chains of varying length and conformation influence the dimerization of appropriately functionalized DNA origami structures.^34^ Origami tiles were modified with fluorous tails of increasing fluorous content [−(CF_2_)_*n*_CF_3_, where *n* ≥ 3], and dimerization was observed only for constructs bearing one (Figure 1B; RF) or more of the longer −(CF_2_)_7_CF_3_ tails (Figure 1B; RF_2_ and RF_4_). Accordingly, the present study focused exclusively on these derivatives. To evaluate their capacity to stabilize triplex formation, we employed a model triplex sequence, in which a pyrimidine-rich 13-nucleotide TFO binds in a parallel orientation to a 13-base pair oligopurine-oligopyrimidine duplex containing four isolated G-C base pairs (Figure S1A).^40–43^ Targeting of such a sequence is strongly pH-dependent and allowed us to assess the stabilizing effect of fluorous interactions at different pH values.

**Figure 2:**
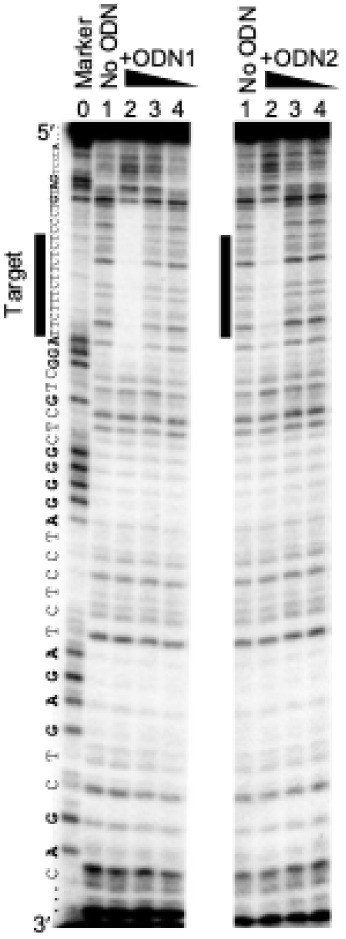
Triplex formation by a fluorous-modified TFO. DNase I cleavage patterns of a 73-mer duplex containing the TFO target sequence in the absence and presence of ODN1 and ODN2 (Figure S1B). Assays were undertaken at pH 5.0 in sodium acetate buffer containing 10 mM MgCl_2_ and the complexes incubated overnight at 4 °C before digestion by the enzyme. Final TFO concentrations were 3, 1, and 0.3 μM in lanes 2, 3, and 4, respectively. The products of the reaction were separated on a 12% denaturing polyacrylamide gel and visualized via phosphorimaging. The TFO target sequence is indicated by the black boxes and was determined by reference to bands in the Maxim–Gilbert “marker” lane (lane 0). The full nucleotide sequence is shown on the left of the gel, and the observed purines are shown in bold.

Our study commenced by comparing the interactions of an unmodified (ODN1) and fluorous-modified TFO (ODN2) with an unmodified duplex sequence embedded within a long duplex fragment (Figures 1B and S1B) using an enzymatic protection assay. The modified 13-mer TFO (ODN2) carried a single perfluoroalkyl (i.e., −(CF_2_)_7_CF_3_) tail incorporated at the 5′-end of an ODN via standard solid-phase methods. Various concentrations of the oligonucleotides were incubated with a ^32^P-labeled 73-mer duplex fragment carrying the embedded target sequence at pH 5.0 in a sodium acetate buffer containing 10 mM MgCl_2_; acidic pH was required to ensure protonation of the four cytosines necessary for TFO binding.^43^ The complexes were digested by DNase I under limiting conditions, and the labeled fragments were separated by denaturing polyacrylamide gel electrophoresis (PAGE).^44^ Cleavage in the presence of the oligonucleotides bound to the DNA resulted in the loss of bands (a “footprint”) due to the TFOs occluding the action of the enzyme at these positions (Figure 2). As predicted, both oligonucleotides give clear footprints (lanes 2–4), and by comparison with a Maxim–Gilbert marker, these are located at the intended target sequence (boxed regions) with an estimated apparent *K*_D_ ∼ 1 μM. Experiments were also performed with a shorter 31-mer duplex (Figure S1B), and the interactions of the ODNs were characterized by an electrophoretic mobility shift assay (EMSA) (Figure S2). Again, both TFOs formed triplexes, as seen by a shift to a slower electrophoretic mobility. These data highlight that attachment of a single perfluorinated tail to the TFO does not significantly impact its interaction with a target site located within a long DNA fragment.

We next evaluated the binding of the ODNs to end-modified duplexes to investigate if positioning fluorous groups in close proximity led to a stabilizing effect. Three different duplex and triplex combinations were assessed, differing by the absence (ODN1) or presence of a fluorous (ODN2) or nonfluorous alkyl tag (ODN3) at the 5′-end of the TFO and/or purine strand of the duplex (DS1–3) (Figure 3A). The alkyl tail was the same length as that of the fluorous tail and was employed to confirm that any stabilization was caused by the fluorous effect rather than hydrophobicity.^34^ The thermal stabilities of the complexes were determined by fluorescence melting experiments using SYBR green I and a Roche LightCycler (Figure 3B).^45^ SYBR green I fluoresces upon binding to both duplex and triplex DNA,^46^ and subsequent melting of the complexes leads to a decrease in fluorescence signal recorded at 522 nm. The midpoint of the melting transition determined from first derivatives is used to determine the melting temperature (*T*_m_) and compare differences in thermal stability of duplex and triplex complexes.^43,44^

**Figure 3:**
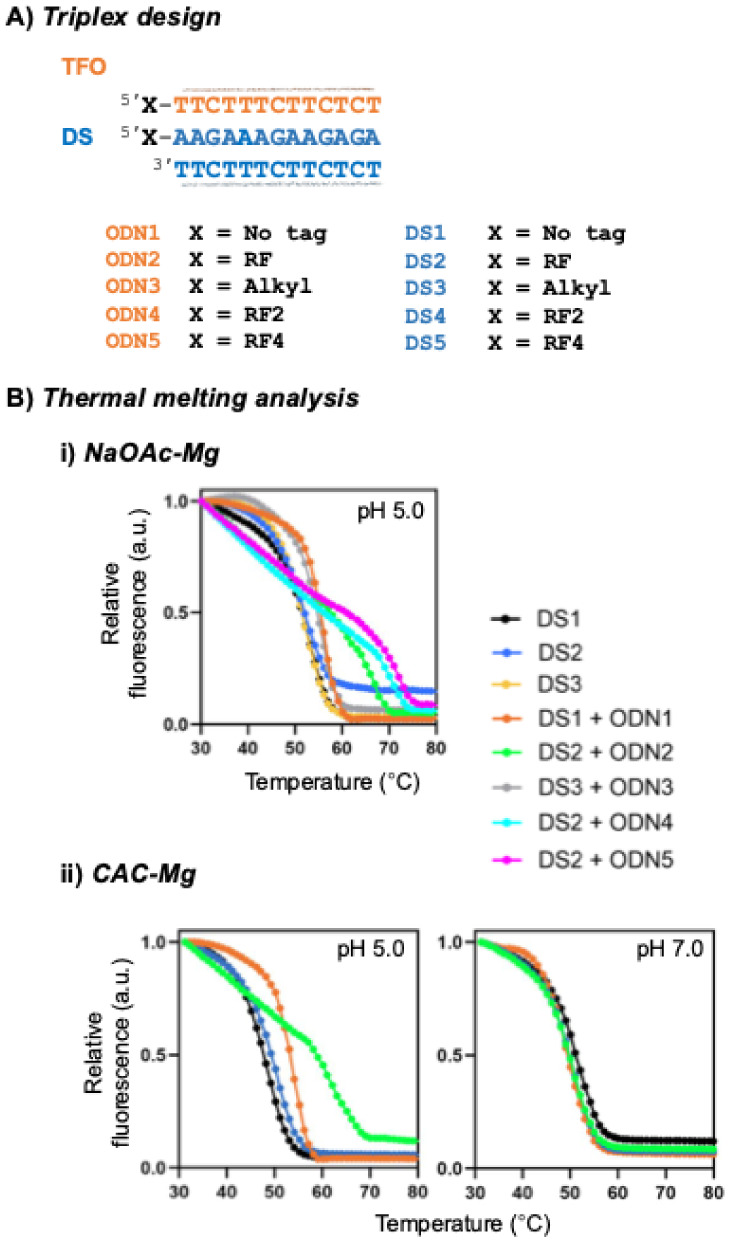
Triplex stabilization by the fluorous effect. (A) Triplex design and (B) fluorescence melting profiles for unmodified and modified triplexes containing single or multiple fluorous or alkyl tags on both the TFO (ODN1–5) and duplex (DS1–3) as indicated. Oligonucleotides were prepared in either (i) sodium acetate (NaOAc-Mg) or (ii) sodium cacodylate (CAC-Mg) buffers containing 10 mM MgCl_2_ at pH 5.0 or pH 7.0 as indicated. The final concentration of the duplex and TFO was 1 μM. Complexes were melted at a rate of 0.2 °C/min in the presence of SYBR green I, and the fluorescence signal was recorded at 522 nm after excitation at 488 nm.

Experiments were carried out at pH 5.0 in a sodium acetate buffer containing 10 mM MgCl_2_, and the melting profiles, first derivatives, and calculated *T*_m_ values are provided in Figure 3B, Figure S3, and Table S1, respectively. In the absence of TFOs, the three duplexes melt in a single transition, yielding *T*_m_ values of ca. 53 °C with no discernible difference in stability (black, blue, and yellow lines). While in the presence of TFOs, the profiles resulted in a stabilization, yielding *T*_m_ values of 55.8 ± 0.4 °C, 67.4 ± 1.5 °C, and 55.8 ± 0.6 °C for the unmodified (orange line), fluorous (green line), and alkyl (gray line) containing triplexes, respectively. Notably, the fluorous-modified triplex was ca. 10 °C more stable than its unmodified or alkyl-modified counterparts, which can be attributed to favorable fluorous interactions. Interestingly, the fluorous-modified complexes produced a less conventional melting profile with a broad decrease in initial fluorescence, likely indicating dissociation and reassociation of the TFO due to its tethering to the adjacent fluorous group on the duplex. Additional experiments confirmed the intermolecular nature of the interactions, and, as expected, the *T*_m_ increased as the concentration of the third strand increased (Figure S4A and Table S2). Furthermore, the modified TFO did not interact with itself or the modified purine strand of the duplex when its pyrimidine partner was absent (Figure S4B). Lastly, UV melting revealed a similar level of stabilization afforded by the attachment of the perfluorinated tails to both the duplex and TFO strands of the triplex (Δ*T*_m_ = 6 °C) in the absence of SYBR green I (Figure S4C).

We then postulated that attachment of multiple perfluorinated tails to the duplex and/or TFO might further improve stability. Indeed, complexes generated with two (ODN4) and four (ODN5) fluorous tails on the TFO and one on the duplex (DS2) produced *T*_m_ values of 72.2 ± 1.7 °C (cyan line) and 73.2 ± 1.6 °C (pink line), respectively (Figure 3B). Compared to the triplex with a single fluorous tail on both the TFO and duplex, a *T*_m_ increase of ca. 5–6 °C was evident. However, further studies with duplexes containing two (DS4) and four (DS5) fluorous tails produced melting profiles (Figure S5A) of lower stability than the unmodified or singly modified duplexes, implying that the tails either reduced duplex stability by clashing with the underlying duplex or caused the samples to aggregate due to fluorous interactions, decreasing their effective concentration. Interestingly, lowering the magnesium concentration (1 mM) overcame this problem but only for the complexes generated with two but not four fluorous groups on the duplex (Figure S5B). Nevertheless, these findings demonstrate fluorous-directed stabilization of intermolecular DNA triplexes can be enhanced using fluorous tails on the oligonucleotides.

To ascertain whether fluorous stabilization is driven by oligonucleotide interactions between the TFO and duplex, we repeated these experiments at neutral pH under conditions known to disrupt intermolecular triplex formation. To make a robust comparison between melting profiles obtained at different pH values, we switched to a sodium cacodylate buffer (CAC-Mg). We measured the stability of the fluorous-tagged complexes at pH 5.0 and pH 7.0, and melting profiles and *T*_m_ values are shown in Figure 3B and Table S3, respectively. Experiments at pH 5.0 revealed that the fluorous-modified triplexes once again showed an increase in stability, with *T*_m_ values of 54.5 ± 1.4 °C and 60.5 ± 0.2 °C for the unmodified (orange line) and modified (green line) triplexes, respectively. However, experiments at pH 7.0 revealed that, as expected, neither the unmodified nor the modified triplexes formed at this pH, and the melting profiles were indistinguishable from the profile for the underlying duplex. This underscores that fluorous stabilization is primarily driven by interactions between the TFO and the duplex.

By contrast to intermolecular complexes, triplexes formed by TFO clamps are less affected by pH, on account of the third strand being tethered to the duplex through pH-independent W–C formation, then associating with the duplex via a pseudo-intramolecular interaction.^3^ We therefore investigated if they produced more stable complexes than their intermolecular equivalents. Perfluorinated tails were attached to both the 5′- and 3′-ends of the ODNs so that upon folding, the fluorous tails are in close proximity (Figure 4A). Two clamp designs were explored. The first positioned the tails at the triplex termini (ODN7) and is comparable in sequence and structure to the intermolecular triplexes described above (Figure 4A). This required attaching the fluorous group to the end of the pyrimidine and not purine strand of the duplex, and consequently, the groups are further apart than in the intermolecular triplex and likely to afford less stabilization. The second was a dumbbell clamp that positioned the two groups internally between the ends of strands that form part of the duplex (ODN9) (Figure 4A).

**Figure 4:**
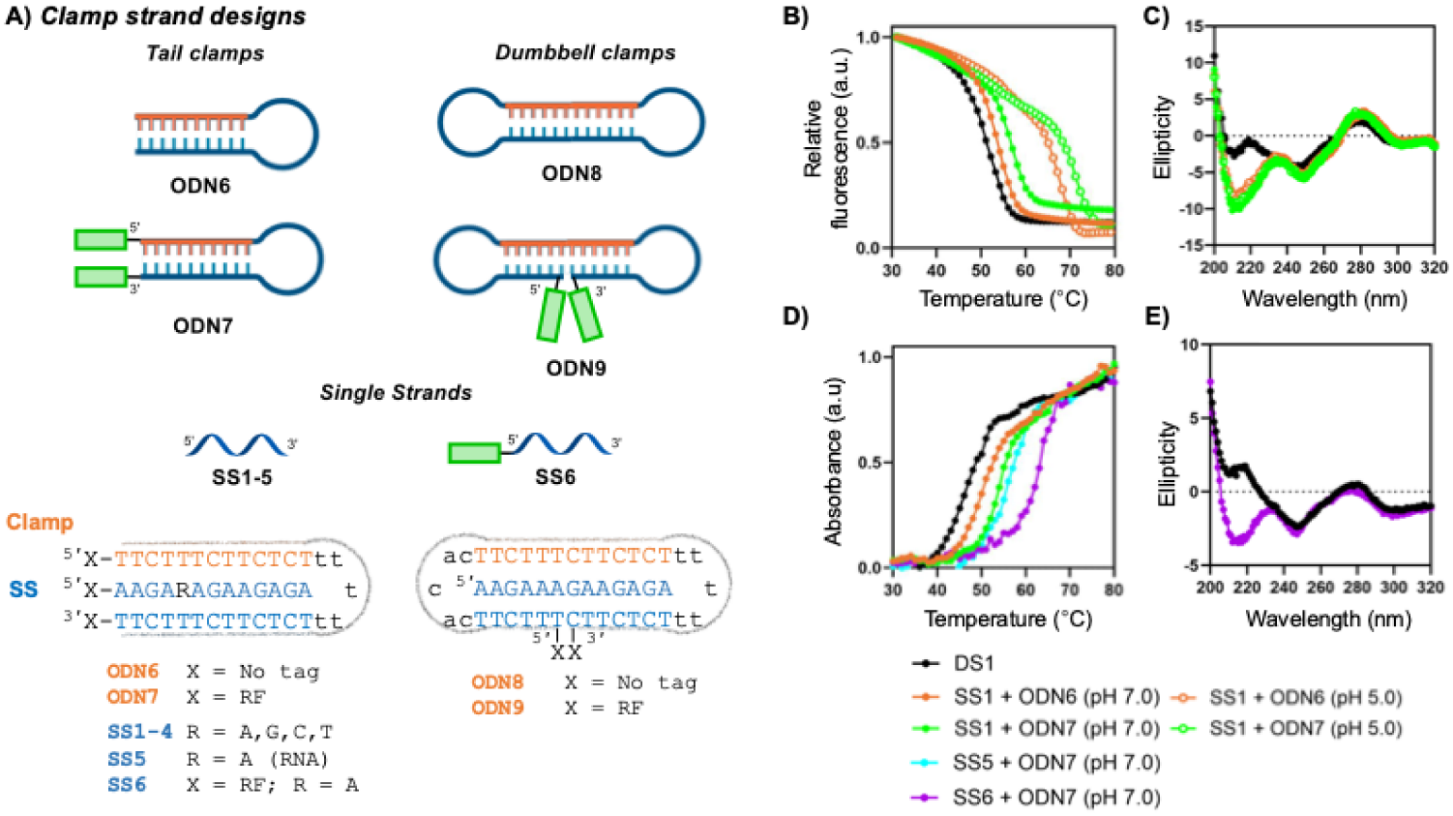
Stable and selective triplex formation by fluorous-modified TFO clamps at neutral pH. (A) Schematic illustration and sequences of intramolecular triplexes investigated in this study. TFO clamp oligonucleotides (ODN) are shown in orange and blue (ODN6–9), single-stranded (SS) sequences are shown in blue (SS1–6), and perfluorinated tails (RF) are shown in green. (B) ODN6 and ODN7 were prepared at pH 5.0 or pH 7.0 in sodium cacodylate buffer containing 10 mM magnesium and investigated for their interaction with single-stranded target SS1. Fluorescence melting profiles for complexes at a final concentration of 1 μM. Complexes were melted at a rate of 0.2 °C/min in the presence of SYBR green I, and the fluorescence signal was recorded at 522 nm after excitation at 488 nm. (C) CD spectra for the same complexes at a final concentration of 5 μM. Oligonucleotides were annealed to 20 °C, and spectra were collected between 320 and 200 nm, with the spectrum of the buffer subtracted. (D) UV melting profiles for selected complexes at a final concentration of 5 μM at pH 7.0. Oligonucleotides were annealed to 20 °C and then melted at a rate of 0.2 °C/min, with the absorbance measured at 260 nm. (E) CD spectra for the interaction of ODN7 with SS6 at a final concentration of 5 μM. Oligonucleotides were annealed to 20 °C, and spectra were collected between 320 and 200 nm, with the spectrum of the buffer subtracted.

Fluorescence melting was used to determine the thermal stabilities at pH 5.0 and 7.0. Analysis of the samples at pH 5.0 revealed triplex assembly by both the unmodified and modified tail clamps, with *T*_m_ values of 67.40 ± 0.03 °C (orange line) and 71.1 ± 0.2 °C (green line), respectively (Figure 4B, Figure S6A and Table S3). As expected, both complexes were more thermally stable than their equivalent intermolecular complex, leading to a ca. 4 °C increase in stability. More importantly, analysis of the complexes at pH 7.0 also revealed successful triplex formation with higher *T*_m_ values than the underlying duplex (Figure 4B and Table S3). Addition of the fluorous group led to an increase in *T*_m_ of ca. 4 °C, shifting the stability of the complex from 55.1 ± 0.6 °C (orange line) to 59.0 ± 0.5 °C (green line). By contrast, attachment of the fluorous group to the dumbbell clamps resulted in a drop in *T*_m_ value at both pH 5.0 and pH 7.0, suggesting triplex formation was destabilized (Figure S6B and Table S3). We also performed circular dichroism (CD) on tail clamps, and analysis of the CD spectra revealed a strong negative peak at 210 nm, which is indicative of an A-type triplex structure (Figure 4C).^47^ These data highlight that the addition of fluorous modifications to TFO clamps stabilizes triplex formation at neutral pH, but only when positioned at the triplex termini.

We next investigated the impact of fluorous stabilization on the sequence selectivity of the tail clamps by assessing their interactions with single-stranded DNA containing mismatched nucleotides at a single central position (Figures 4A, SS2, SS3, and SS4) by fluorescence melting (Figure S7 and Table S4). Interestingly, the fluorous-modified clamp exhibited enhanced sequence selectivity, displaying a c*a.* 13 °C reduction in *T*_m_ between the triplex with the most stable (T:A-T) and the second most stable (T:G-T) triplets, compared to only a ca. 5 °C decrease observed with the same triplets for the unmodified tail clamp. These findings indicate that the fluorous tails do not compromise the sequence selectivity of oligonucleotide interactions.

Finally, we explored if the fluorous-modified tail clamp could bind to other single-stranded oligonucleotides. We first investigated the interaction of the modified tail clamp with an equivalent RNA oligonucleotide (Figures 4A, SS5) and assessed binding by UV melting analysis alongside the DNA-only complex as a control (Figure 4D and Table S3). Both complexes exhibited single melting transitions, with *T*_m_ values of 54 and 55 °C, for the DNA (green line) and RNA (cyan line) strands, respectively. Additionally, we assessed the interaction of the clamps with a strand carrying a single perfluorinated group at the 5′-end (Figures 4A, SS6). The resulting complex, which positions three tails in close proximity, exhibited a *T*_m_ of 63 °C (purple line) and is more than 8 °C higher than the complex assembled with two modifications. CD analysis confirmed the formation of a DNA triplex, with a negative peak at 220 nm (Figure 4E).

In summary, this study showed that attachment of single or multiple perfluorinated tails to the 3′ and/or 5′ end of TFO and TFO clamps enhances the thermal stability of the triplex complexes. TFO clamps remain stable at neutral pH without compromising the selectivity of oligonucleotide interactions. The most stable triplex was generated with ODNs containing perfluorinated tails at the ends of all three triplex strands. The observed level of stabilization is comparable to that observed in triplexes containing one or two stabilizing base or sugar modifications in the TFO, such as locked nucleic acids (LNA).^7^ This clamping strategy is simple to implement by solid-phase synthesis and could find broad utility as a facile strategy to deliver TFO cargo into living cells or as an auxiliary tool for the hierarchical assembly of DNA nanostructures. Moreover, the same approach could be utilized to stabilize other nucleic acid structures, such as i-motifs or G-quadruplexes, for either their study or application.

## Experimental Section

### Oligonucleotides and Perfluorinated Conjugates

Oligonucleotide sequences used in this study are shown in Figures 2, 3, 4, S1, and S5 and were synthesized in-house or purchased from Sigma-Aldrich.

Oligodeoxyribonucleotides (ODN) were synthesized according to standard solid-phase oligonucleotide-synthesis protocols on an ABI 392 DNA/RNA synthesizer on a 1 μM scale. Coupling efficiency was monitored after removal of the dimethoxytrityl (DMTr) 5′-OH protecting groups. Controlled pore glass (CPG, 1000Å/110 μm) supports loaded with standard nucleosides and standard phosphoramidites were purchased from LINK-LGC Biosearch Technologies.

Special branched phosphoramidite **1** (0.1 M solution in CH_3_CN), alkyl phosphoramidite **2**, and single fluorous phosphoramidite **3** (0.2 M solution in CH_3_CN) were introduced at the 5′ position using a 1 μM CE cycle. When incorporating multiple fluorous tags using phosphoramidite **3** (0.2 M solution in CH_3_CN) after branched phosphoramidite **1**, a modified cycle was used with longer coupling times (double coupling, 3 min each) (Scheme 1).

**Scheme 1:**
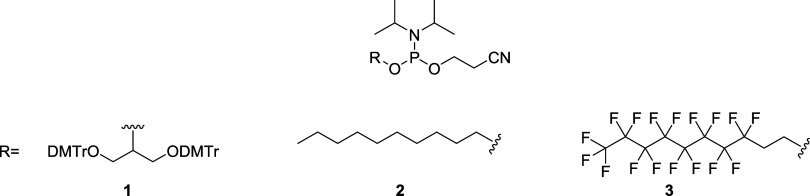
Structure of the Phosphoramidites Used in the Synthesis of the ODNs

#### Cleavage and Deprotection Procedure

ODNs were cleaved from the solid support and deprotected in 28% aqueous ammonium hydroxide solution for 4 h at 55 °C or overnight at room temperature. The crude product solution was separated from the solid support and concentrated under reduced pressure at 30 °C in a speed vacuum concentrator. This crude solid was resuspended in 1 mL of Millipore water and purified by HPLC.

#### HPLC Purification

Analytical RP-HPLC was performed at rt on an ULTIMAT 3000 Instrument (DIONEX) (Figures S8–S17). UV absorbance was measured using a photodiode array detector at 260 nm. A Clarity Oligo RP C18 (10 × 250 mm, 5 μm) column was used for semiprep RP-HPLC. Solvent system: Buffer A, 0.1 M Triethylammonium acetate (TEAA) in H_2_O, pH 7.4; Buffer B, 0.1 M TEAA, pH 7.4, 80% MeCN and 20% H_2_O. Flow rate: 3.0 mL/min.

Gradient Profile:


**Time (min)**

**%A**

**%B**

**0**
9010
**3**
9010
**38**
0100
**50**
0100
**51**
9010
**60**
9010

### DNA Fragment for DNase I Protection Assay

The 73-mer DNA fragment containing the embedded TFO target sequence used in DNase I footprinting experiments is shown in Figure S1B. To amplify the fragment, a recombinant plasmid was transformed into competent *Escherichia coli* TG2 cells, and the plasmid was isolated using a Qiagen Miniprep kit. The plasmid was then digested with *Hin*dIII and SacI (New England Biolabs) and radiolabeled at the 3′-end of the *Hin*dIII site using the exo-Klenow fragment (New England Biolabs) and [α-^32^P]­dATP (PerkinElmer). The fragment was separated from the remainder of the plasmid on an 8% (w/v) nondenaturing polyacrylamide gel. After elution, the fragment was dissolved in 10 mM tris–HCl at pH 7.0 to yield approximately 10 c.p.s./μL, as determined by a hand-held Geiger counter (corresponding to <10 nM DNA).

### DNase I Protection Assay

Samples were prepared by mixing 1.5 μL radiolabeled DNA with 3 μL TFO dissolved in 50 mM sodium acetate containing 10 mM MgCl_2_ at pH 5.0. Final TFO concentrations varied between 0.1 and 10 μM, and the complexes were left to equilibrate overnight at 4 °C. DNase I digestion was performed by adding 2 μL DNase I (typically 0.01 U/ml) dissolved in 20 mM NaCl containing 2 mM MgCl_2_ and 2 mM MnCl_2_. The reaction was then stopped after 1 min by adding 4 μL of 80% formamide containing 10 mM EDTA, 10 mM NaOH, and 0.1% (w/v) bromophenol blue. The cleavage products were then separated on a 12% (w/v) polyacrylamide gel containing 8 M urea. Before loading, samples were heated to 100 °C for 3 min and then rapidly cooled on ice. Polyacrylamide gels were run at 1500 V for ∼2 h, fixed in 10% (v/v) acetic acid, transferred to Whatman 3MM paper, and dried under vacuum at 86 °C for 1 h. The dried gels were subjected to phosphorimaging using a Molecular Dynamics Typhoon PhosphorImager.

### DNA Fragment for EMSA Assay

The 31-mer DNA fragment containing the embedded TFO target sequence used in EMSA experiments is shown in Figure S1B. The sequence was radiolabeled at the 5′-end using polynucleotide kinase (New England Biolabs) and [γ-^32^P]­ATP (PerkinElmer). The fragment was separated from unincorporated label on a 20% (w/v) nondenaturing polyacrylamide gel. After elution, the fragment was dissolved in 10 mM tris–HCl at pH 7.0 to yield approximately 10 c.p.s./μL, as determined by a hand-held Geiger counter (corresponding to <100 nM DNA).

### Electrophoretic Mobility Shift Assay (EMSA)

Oligonucleotides were prepared at pH 5.0 in 50 mM sodium acetate containing 10 mM MgCl_2_. The final duplex concentration was <100 nM, and the TFO concentrations varied between 0.01 and 10 μM. Samples were prepared in a total volume of 20 μL. The complexes were initially heated to 90 °C for 3 min, gradually cooled to room temperature, and then allowed to equilibrate at 20 °C for >16 h. Samples were separated on nondenaturing 20% (w/v) polyacrylamide gels, run at 400 V for approximately 2 h in 40 mM tris-acetate running buffer containing 10 mM MgCl_2_. They were then fixed in 10% (v/v) acetic acid, transferred to Whatman 3MM paper, and dried under vacuum at 86 °C for 1 h. The dried gels were subjected to phosphorimaging using a Molecular Dynamics Typhoon PhosphorImager.

### Fluorescence Melting with SYBR Green I

Thermal melting profiles for the duplex and triplexes were determined using SYBR green I (Thermo Fisher) and a Roche LightCycler.^45^ Oligonucleotides were prepared in either 50 mM sodium acetate containing 10 mM MgCl_2_ at pH 7.0, or 10 mM sodium cacodylate containing 10 mM MgCl_2_ at the appropriate pH. The final concentration of the duplex was 1 μM, and final TFO concentrations varied between 0.1 and 10 μM depending on the experiment. Samples were prepared in a total volume of 20 μL. Fluorescence emission from SYBR green I was recorded at 522 nm after excitation at 488 nm. Fluorescence profiles were obtained for both annealing and melting of the complexes between 30 and 95 °C at a ramp rate of 0.2 °C/min. Melting temperatures (*T*_m_) were determined from the first derivatives of the profiles using the software provided with the machine and typically differed by <0.5 °C between experiments.

### Circular Dichroism

CD spectra of the duplexes and triplexes were determined using a JASCO J-710 spectropolarimeter. Oligonucleotides were dissolved in 10 mM sodium cacodylate containing 10 mM MgCl_2_ at the pH indicated. The final concentration of the triplex was 10 μM in a total volume of 300 μL. The complexes were first heated to 95 °C for 5 min, slowly cooled to room temperature, and then left at 20 °C to equilibrate for 16 h. Spectra were collected between 320 and 200 nm at 100 nm/min, with a 1 s response time and 1 nm bandwidth in Hellma synthetic quartz cuvettes with a 1 mm path length. Each spectrum was accumulated five times, smoothed, and the buffer spectrum was subtracted.

### UV Melting

UV melting experiments on the complexes formed by tail clamp oligonucleotides were carried out using a Shimadzu UV-2600 UV–vis spectrophotometer with a TMSPC-8 temperature controller. Absorbance was determined in a Shimadzu 8 Series Micro Cell quartz block with a 10 mm path length, monitoring at 260 nm. The oligonucleotides were prepared in 10 mM sodium cacodylate buffer containing 10 mM MgCl_2_ at pH 7.0. Melting experiments were carried out in a total volume of 100 μL and contained 5 μM clamp oligonucleotide and 10 μM R strand. The complexes were first heated to 95 °C for 5 min, slowly cooled to room temperature, and then left at 4 °C to equilibrate for 16 h. UV profiles were obtained for melting of the complexes between 22 and 90 °C at a ramp rate of 0.2 °C/min. Recordings were taken at 1 °C intervals. *T*_m_ values were determined from the first derivatives of the melting profiles using the software provided with the machine and usually differed by less than 1 °C.

## Supplementary Material



## Data Availability

The data is available throughout the manuscript and supporting files.

## Notes

Oligonucleotide sequences under study; purification protocols and oligonucleotide characterization; additional gel electrophoresis experiments and thermal melting data and analysis (PDF)
