# Supplementary material for: Fluorous-Directed Clamping Stabilizes Triple-Helical DNA
Source: ACS Omega. 2026 Jun 1;11(23):33983–90. doi: 10.1021/acsomega.6c00803 (PMC13280840; doi:10.1021/acsomega.6c00803)
Supplement: Supplementary file 1 [file ao6c00803_si_001.pdf]

Supplementary Information

Fluorous-Directed Clamping Stabilises Triple-Helical DNA

Andrea Taladriz-Sender,[a,d] Michael Brazzill,[b] Jamie M. Withers,[a,d] Alasdair W. Clark,[c] Glenn A. Burley,[a,d*] and David A. Rusling[b*]

[a]Department of Pure Applied Chemistry, Thomas Graham Building, 295 Cathedral Street, University of Strathclyde, Glasgow, G1 1XL.

[b]School of Medicine, Pharmacy and Biomedical Sciences, University of Portsmouth, Portsmouth, PO1 2DT, UK

[c]James Watt School of Engineering, Advanced Research Centre, University of Glasgow, Glasgow, G11 6EW, UK.

[d] Strathclyde Centre for Molecular Bioscience, University of Strathclyde, Glasgow G1 1XQ, U.K.

*Corresponding authors: david.rusling@port.ac.uk and glenn.burley@strath.ac.uk

A)

	Sequence	X =
ODN1	$^{5'}$TTCTTTCTTCTCT$^{3'}$	-
ODN2	$^{5'}$X-TTCTTTCTTCTCT$^{3'}$	$-C_2H_4C_8F_{17}$
ODN3	$^{5'}$X-TTCTTTCTTCTCT$^{3'}$	$-C_{10}H_{21}$
ODN4	$^{5'}$X-TTCTTTCTTCTCT$^{3'}$	$-(PO_4C_2H_4C_8F_{17})_2$
ODN5	$^{5'}$X-TTCTTTCTTCTCT$^{3'}$	$-(PO_4C_2H_4C_8F_{17})_4$
DS1	$^{5'}$AAGAAAGAAGAGA$^{3'}$<br>$^{3'}$TTCTTTCTTCTCT$^{5'}$	-
DS2	$^{5'}$X-AAGAAAGAAGAGA$^{3'}$<br>$^{3'}$TTCTTTCTTCTCT$^{5'}$	$-C_2H_4C_8F_{17}$
DS3	$^{5'}$X-AAGAAAGAAGAGA$^{3'}$<br>$^{3'}$TTCTTTCTTCTCT$^{5'}$	$-C_{10}H_{21}$
DS4	$^{5'}$X-AAGAAAGAAGAGA$^{3'}$<br>$^{3'}$TTCTTTCTTCTCT$^{5'}$	$-(PO_4C_2H_4C_8F_{17})_2$
DS5	$^{5'}$X-AAGAAAGAAGAGA$^{3'}$<br>$^{3'}$TTCTTTCTTCTCT$^{5'}$	$-(PO_4C_2H_4C_8F_{17})_4$
ODN6	$^{5'}$TTCTTTCTTCTCTTTTTTTTCTCTTCTTTCTT$^{3'}$	-
ODN7	$^{5'}$X-TTCTTTCTTCTCTTTTTTTTCTCTTCTTTCTT-X$^{3'}$	$-C_2H_4C_8F_{17}$
ODN8	$^{5'}$TTTCTTCACACTTCTTTCTTCTCTTTTTTTTCTCTTC$^{3'}$	-
ODN9	$^{5'}$X-TTTCTTCACACTTCTTTCTTCTCTTTTTTTTCTCTTC-X$^{3'}$	$-C_2H_4C_8F_{17}$
SS1	$^{5'}$AAGAAAGAAGAGA$^{3'}$	-
SS2	$^{5'}$AAGAGAGAAGAGA$^{3'}$	-
SS3	$^{5'}$AAGATAGAAGAGA$^{3'}$	-
SS4	$^{5'}$AAGACAGAAGAGA$^{3'}$	-
SS5	$^{5'}$AAGAAAGAAGAGA$^{3'}$(RNA)	-
SS6	$^{5'}$X-AAGAAAGAAGAGA$^{3'}$	$-C_2H_4C_8F_{17}$

B)

```
5'-*GAGCAGCCTAAGAAAGAAGAGAGGGACATCA
    CTCGTCGGATTCTTTCTTCTCTCCCTGTAGT-5'

5'-AGCTTGCATGCCTGCAGGTCGACTCTAGAGGATCCCCGAGCAGCCTAAGAAAGAAGAGAGGGACATCAGGGTACCGAGCT
     *AACGTACGGACGTCCAGCTGAGATCTCCTAGGGGCTCGTCGGATTCTTTCTTCTCTCCCTGTAGTCCCATGGC-5'
```

Figure S1: (A) Sequences of the oligonucleotides used in this study (B) Sequence of the 31-bp or 73-bp duplex fragments containing the embedded TFO target sequence used in the DNase I protection assay and EMSA experiments, respectively. The target sequence is boxed, and the strands were radiolabelled at the positions indicated by the asterisk.

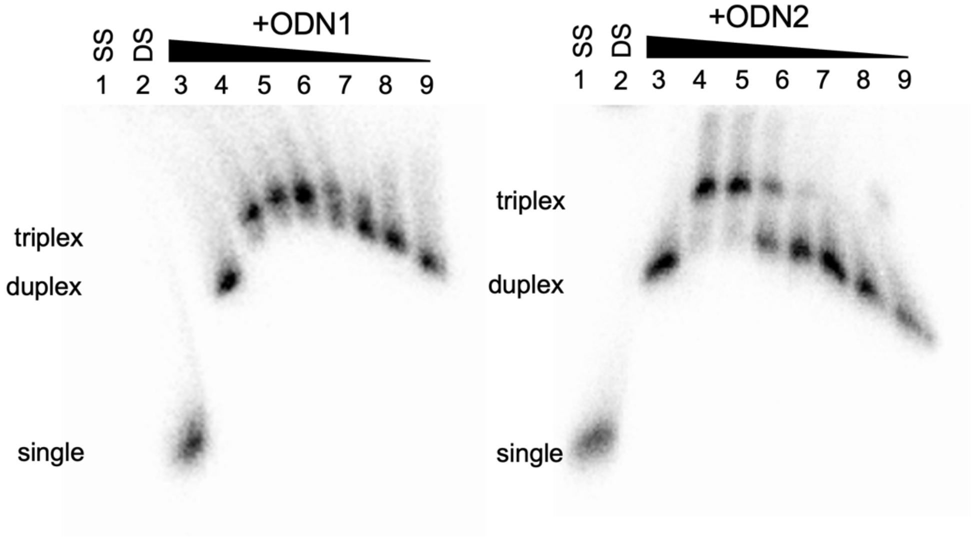

Figure S2: Triplex formation between a fluorous modified TFO and unmodified duplex. EMSA of a 31-mer duplex containing the TFO target sequence in the absence and presence of ODN1 and ODN2 (Fig S1B). Assays were undertaken at pH 5.0 in sodium acetate buffer containing 10 mM $MgCl_2$ and the complexes incubated overnight at 4 °C. The final duplex concentration was <50 nM and the final TFO concentration was varied between 10 and 0.01 μM (lanes 4-9). Complexes were separated on a 20% non-denaturing polyacrylamide gel in tris-acetate running buffer lacking EDTA and containing 10 mM magnesium and visualised *via* phosphorimaging.

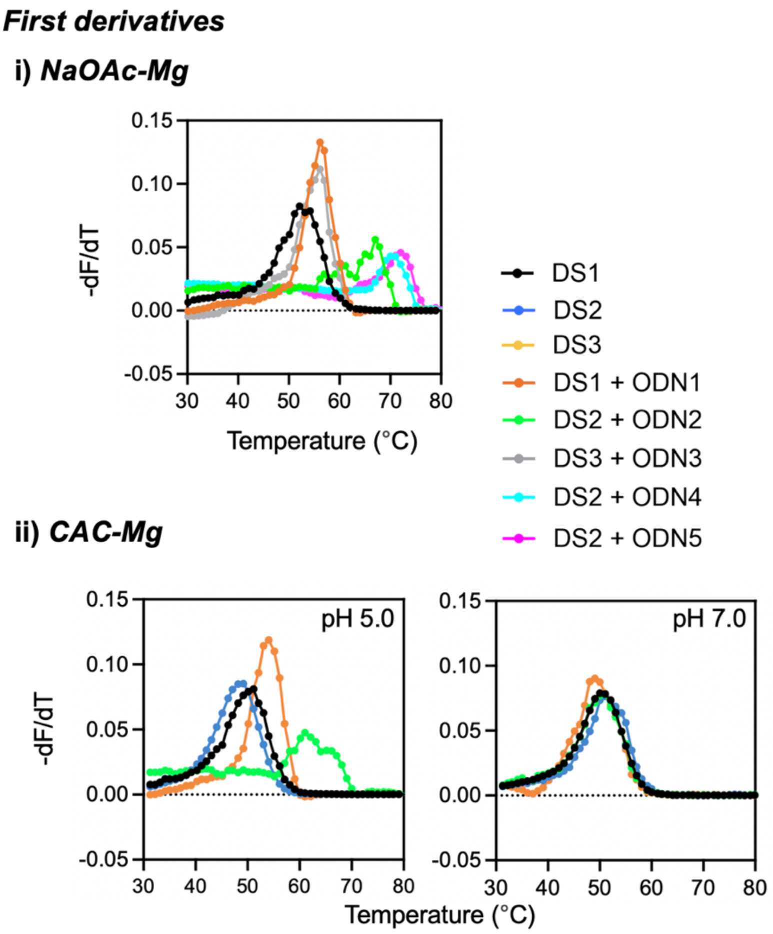

Figure S3. Triplex stabilisation by the fluorous effect (first derivatives). First derivatives of fluorescence melting profiles for unmodified and modified triplexes containing single or multiple fluorous or alkyl tags on both the TFO (ODN1-5) and duplex (DS1-3). Oligonucleotides were prepared in either (i) sodium acetate (NaOAc-Mg) or (ii) sodium cacodylate (CAC-Mg) buffers containing 10 mM $MgCl_2$ at pH 5.0 or pH 7.0. The final concentration of the duplex and TFO was 1 µM. Complexes were melted at a rate of 0.2 °C/min in the presence of SYBR green I and fluorescence signal recorded at 522 nm after excitation at 488 nm. First derivatives were used to determine T_m values.

Table S1: T_m values and standard errors (°C) for unmodified, perfluorinated, and alkyl triplexes determined at pH 5.0 in 10 mM sodium acetate buffer containing 10 magnesium. Representative fluorescence melting profiles are shown in Figure 3 and first derivatives in Figure S3.

	Modification	DS only	+ODN
DS1 **+ODN1**	Unmodified	52.7 ± 0.3	55.8 ± 0.4
DS2 **+ODN2**	Fluorous	53.2 ± 0.3	67.4 ± 1.5
DS3 **+ODN3**	Alkyl	52.0 ± 0.7	55.8 ± 0.6

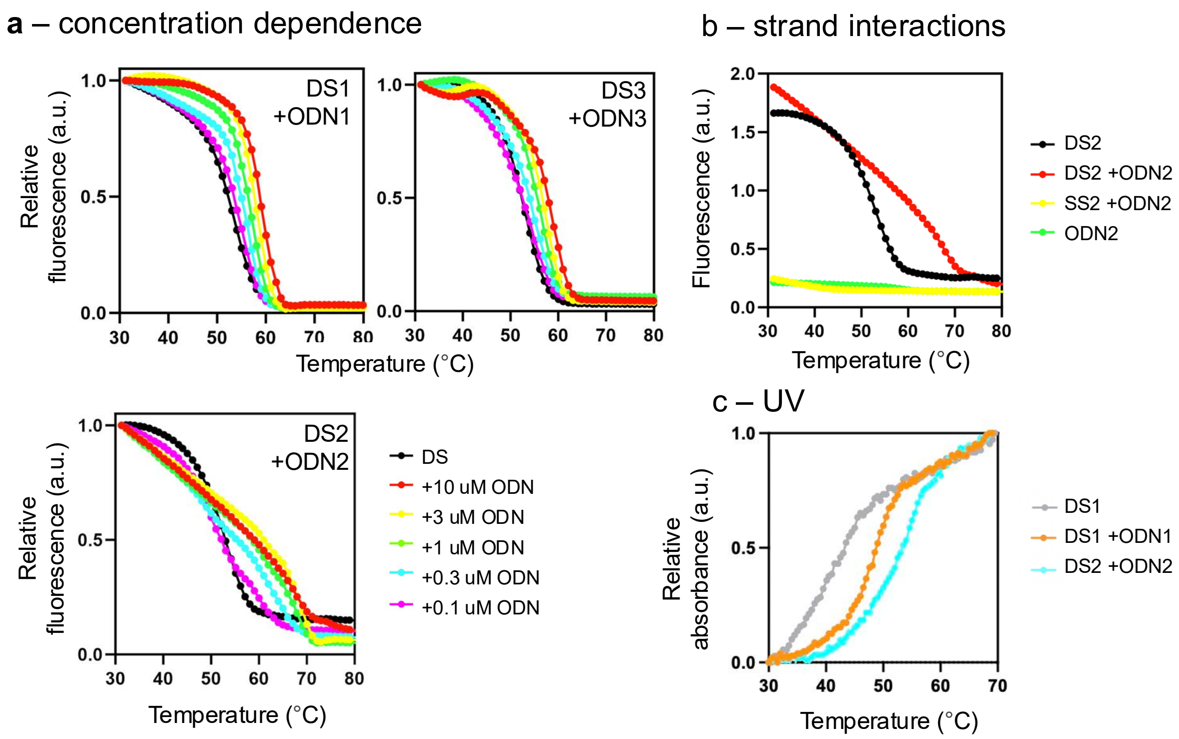

Figure S4: Control experiments. (a) Fluorescence melting profiles for three different duplex and triplex combinations, differing by the absence (ODN1) or presence of a fluorous (ODN2) or non-fluorous alkyl tag at the 5′-end of the TFO (ODN3) and purine strand of the duplex (DS1-3) Oligonucleotides were prepared at pH 5.0 in sodium acetate buffer containing 10 mM $MgCl_2$. The final concentration of the duplex was 1 μM, whilst the final concentration of the TFO was varied between 10 μM and 0.1 μM as indicated. (b) Fluorescence recordings for different strand combinations containing the fluorous-modified ODN1. Oligonucleotides were at pH 5.0 prepared in a sodium acetate buffer containing 10 mM $MgCl_2$. The final concentration of the duplex and TFO stands was 1 and 10 μM, respectively. Complexes were melted at a rate of 0.2 °C/min in the presence of SYBR green and the fluorescence signal recorded at 522 nm after excitation at 488 nm. (C) UV melting profiles for selected complexes at a final concentration of 5 μM. Oligonucleotides were prepared at pH 5.0 in sodium acetate buffer containing 10 mM $MgCl_2$ and annealed to 20 °C. Samples were then melted at a rate of 0.2 °C/min and the absorbance measured at 260 nm.

Table S2: T_m values (°C) for unmodified, perfluorinated, and alkyl triplexes with different concentrations of TFO at pH 5.0 in 10 mM sodium acetate buffer containing 10 mM $MgCl_2$. Representative fluorescence melting profiles are shown in Figure S3.

	Modification	DS only	+ ODN				
			10 µM	3 µM	1 µM	0.3 µM	0.1 µM
DS1 **+ODN1**	Unmodified	53.5	59.2	58.1	57.1	55.9	55.2
DS2 **+ODN2**	Fluorous	53.2	66.9	68.0	67.4	62.3	60.3
DS3 **+ODN3**	Alkyl	53.0	58.9	57.5	56.1	55.2	54.2

a – multiple Ft on duplex (10 mM Mg^{2+})

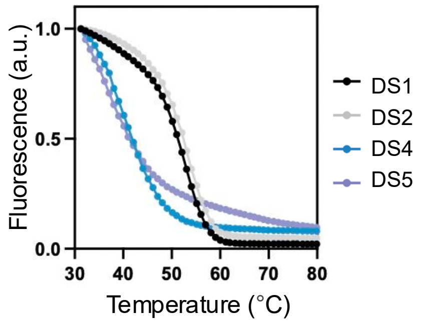

b – multiple Ft on duplex (1 mM Mg^{2+})

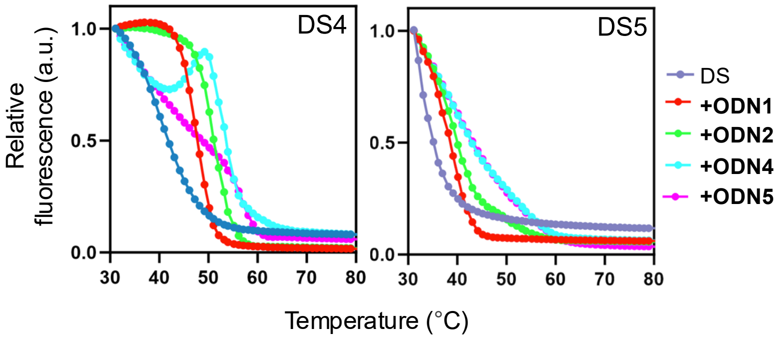

Figure S5: Increasing fluorous density on the duplex leads to aggregation of the complexes at high magnesium concentrations. Fluorescence melting profiles for unmodified and modified duplexes (a) and triplexes (b) containing different numbers of fluorous groups attached to the TFO and/or duplex. Oligonucleotides were prepared at pH 5.0 in sodium acetate buffer containing 10 or 1 mM $MgCl_2$. The final concentration of the duplex and TFO was 1 µM. Complexes were melted at a rate of 0.2 °C/min in the presence of SYBR green and the fluorescence signal recorded at 522 nm after excitation at 488 nm. The T_m values at 1 mm $MgCl_2$ for DS4 that contained two RF groups was 42 °C, and increased to 47.8 °C, 51.4 °C, 53.4 °C and 57.8 °C in the presence of ODN1, ODN2, ODN4, and ODN5, respectively (left panel). It was not possible to measure the T_m values for the samples containing four RF groups on the duplex (right panel).

Table S3: T_m values and standard errors (°C) for unmodified and fluorous-modified complexes determined at pH 5.0 and 7.0 in sodium cacodylate buffer containing 10 mM $MgCl_2$. ΔT_m values compare the modified and equivalent unmodified complexes. Representative melting profiles are shown in Figure 3B, 4B, 4D and S4C.

		Fluorescence melting				UV melting			
Complex	Type	pH 5.0	ΔT_m	pH 7.0	ΔT_m	pH 5.0	ΔT_m	pH 7.0	ΔT_m
DS1	Inter	47.5 ± 0.6		50.8 ± 0.2		42		47	
DS1 **+ODN1**	Inter	54.5 ± 1.4		50.7 ± 0.1		49			
DS2	Inter	49.0 ± 0.8		51.8 ± 0.1					
DS2 **+ODN2**	Inter	60.5 ± 0.2	**+6.0**	51.6 ± 0.3	**+0.9**	55	**+6**		
SS1 **+ODN6**	Clamp	67.4 ± 0.1		55.1 ± 0.6				49	
SS1 **+ODN7**	Clamp	71.1 ± 0.2	**+3.7**	59.0 ± 0.5	**+3.9**			54	**+5**
SS1 **+ODN8**	Clamp	61.4 ± 0.3		54.0 ± 0.1					
SS1 **+ODN9**	Clamp	58.2 ± 0.2	**-3.2**	44.9 ± 0.1	**-9.1**				
SS5 **+ODN7**	Clamp							55	**+6**
SS6 **+ODN7**	Clamp							63	**+14**

A) *ODN6/ODN9*

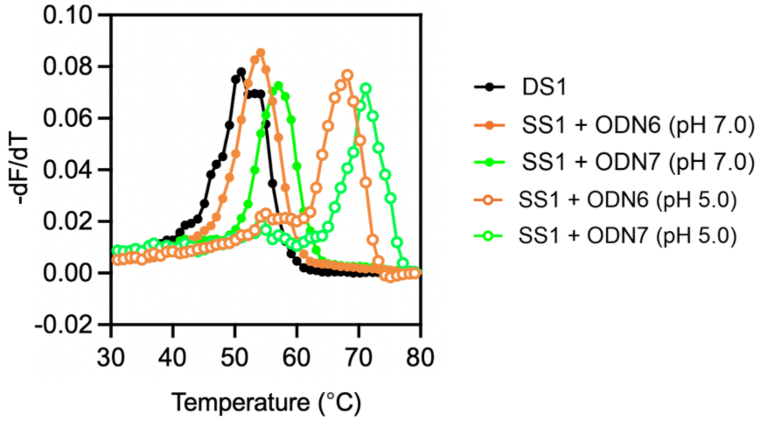

B) *ODN8/ODN9*

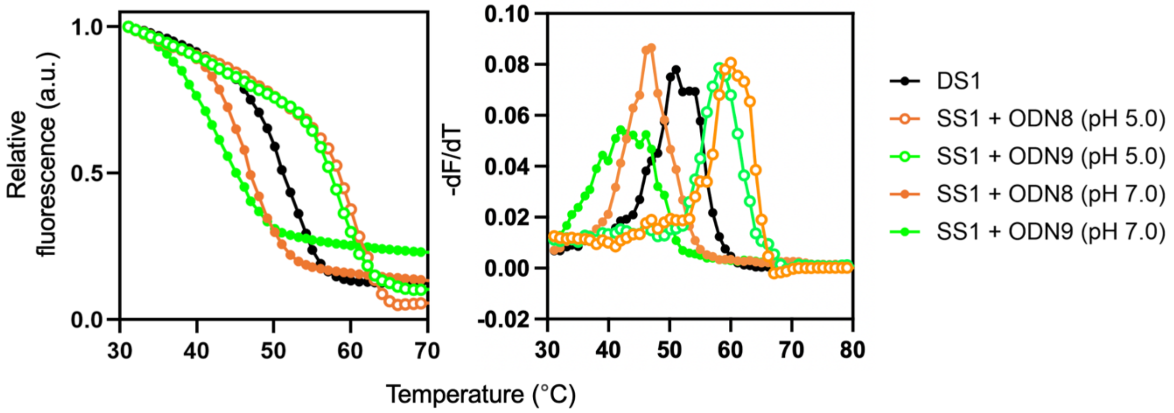

Figure S6: Stabilisation of triplex clamps by the fluorous effect (first derivatives). (A) Stable and selective triplex formation by fluorous-modified TFO clamps at neutral pH (first derivatives). ODN6 and ODN7 were prepared at pH 5.0 or pH 7.0 in sodium cacodylate buffer containing 10 mM magnesium and investigated for their interaction with single-stranded target SS1. Fluorescence melting profiles for complexes at a final concentration of 1 µM. Complexes were melted at a rate of 0.2 °C/min in the presence of SYBR green I and the fluorescence signal recorded at 522 nm after excitation at 488 nm. First derivatives were used to determine T_m values. (B) Triplex formation by fluorous-modified dumbbell clamps is destabilising at neutral pH. TFO clamp oligonucleotides ODN8 and ODN9 and investigated for their interaction with SS1. Fluorescence melting profiles for dumbbell clamp oligonucleotides ODN8 and ODN9 with SS1. The oligonucleotides were prepared at a final concentration of 1 µM at pH 5.0 or pH 7.0 in sodium cacodylate buffer containing 10 mm $MgCl_2$. Complexes were melted at a rate of 0.2 °C/min in the presence of SYBR green I and the fluorescence signal recorded at 522 nm after excitation at 488 nm. First derivatives were used to determine T_m values.

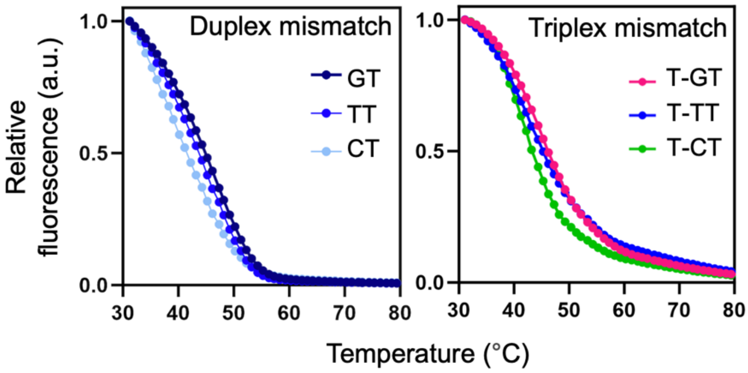

Figure S7: Triplex and duplex mismatches dramatically decrease tail clamp stability at neutral pH. Fluorescence melting profiles for triplexes assembled from ODN7 and SS2-4. Oligonucleotides were prepared in sodium cacodylate buffers containing 10 mM $MgCl_2$. The final concentrations of the single-stranded targets and clamps were 1 µM and 2 µM, respectively. Complexes were melted at a rate of 0.2°C/min in the presence of SYBR green and the fluorescence signal recorded at 522 nm after excitation at 488 nm.

Table S4: T_m values (°C) for unmodified and fluorous-modified TFO clamps with single-stranded sequences containing a mismatched nucleotide. Experiments were undertaken at pH 7.0 in a sodium cacodylate buffer containing 10 mM magnesium. Representative fluorescence melting profiles are shown in Figure S6.

(mismatch)	DS	ΔT_m	+ ODN6	ΔT_m	+ ODN7	ΔT_m
SS1[a] (AT)	50.6		54.2		58.0	
SS2 (GT)	47.1	**-3.5**	49.3	-4.9	45.1	**-12.9**
SS3 (TT)	46.2	**-4.4**	46.6	-7.6	43.0	**-15**
SS4 (CT)	39.8	**-10.8**	43.2	-11	42.1	**-15.9**

[a]data from above

Oligonucleotide analytical methods and characterisation

Purity Analysis Method A

Thermo Scientific Vanquish Flex System

Column Specifications: XBridge™ Premier Oligo column, BEH C18, 2.1 × 50 mm, 2.5 µm.

Column Temperature: 60 °C

Mobile Phase A: 50mM HFIP, 5 mM DIPEA in H_2O

Mobile Phase B: 50mM HFIP, 5 mM DIPEA in MeCN: H_2O (1:1)

Flow rate: 0.6 mL/min

Injection Volume: 2 µL

Gradient:

Time (min)	B%
**0 – 6**	10 – 100
**6 – 7**	100
**7 – 7.5**	100-10
**7.5 – 9.5**	10

Purity Analysis Method B

Waters ACQUITY Premier UPLC

Column Specifications: Sinergy™ Fusion RP, 50Å, 2 × 50 mm, 2.5 µm.

Column Temperature: 60 °C

Mobile Phase A: 200 mM Ammonium Acetate in H_2O pH 4.5

Mobile Phase B: 200 mM Ammonium Acetate in MeOH

Flow rate: 0.3 mL/min

Injection Volume: 2 µL

Gradient:

Time (min)	B%
**0 – 15**	5 – 100
**15 – 17**	100
**17 – 17.1**	100-5
**17.1 – 20**	5

Purity Analysis Method C

Thermo Scientific Vanquish Flex System

Column Specifications: XBridge™ Premier Oligo column, BEH C18, 2.1 × 50 mm, 2.5 µm.

Column Temperature: 50 °C

Mobile Phase A: 0.1 M Triethylammonium acetate (TEAA) in H_2O, pH 7.4

Mobile Phase B: 0.1 M TEAA, MeCN: H_2O (8:2), pH 7.4

Flow rate: 0.6 mL/min

Injection Volume: 2 µL

Gradient:

Time (min)	B%
**0 – 6**	10 – 100
**6 – 7**	100
**7 – 7.5**	100-10
**7.5 – 9.5**	10

Mass Spectrometry Analysis Method

LC-MS analysis of oligonucleotides was performed on a Waters BioAccord consisting of a Waters ACQUITY Premier (QSM, SM-FTN, CH-A and TUV detector) and a RDa detector. Acquired mass range was set to high (400 – 15000 m/z) with a negative polarity and a scan rate of 2 Hz. Cone voltage was set to 40 V. Capillary voltage was set to 0.80 kV and the desolvation temperature to 450 °C. Flow was only directed to the Rda detector between 0.5 – 5.5 mins. Intelligent data capture was set to on. Absorbance was collected at 260 nm using a data rate of 20 Hz. Column temperature was maintained at 40 °C. The flow rate was set to 0.4 ml/min. Buffer C was 50 mM ammonium acetate in H_2O and buffer D was MeOH/H_2O (9:1). An ACQUITY UPLC Protein BEH C4, 300 Å, 1.7 µm, 2.1 mm × 50 mm column was used to separate oligonucleotides with the following gradient:

Time (min)	C (%)	D (%)
0.0	99	1.0
0.5	99	1.0
1.0	95	5.0
1.5	90	10
5.0	75	25
5.1	5	95
6.0	5	95
6.1	99	1.0
7.5	99	1.0

Table S5: Purity and masses of oligonucleotides synthesised

	Sequence	X	Theoretical Mass	Observed Mass	HPLC Purity
ODN2	5'X-TTCTTTCTTCTCT3'	$-C_2H_4C_8F_{17}$	4356.6067	4356.590	96%
ODN3	5'X-TTCTTTCTTCTCT3'	$-C_{10}H_{21}$	4050.7669	4050.740	97%
ODN4	5'X-TTCTTTCTTCTCT3'	$-(PO_4C_2H_4C_8F_{17})_2$	5036.5725	5036.573	99%
ODN5	5'X-TTCTTTCTTCTCT3'	$-(PO_4C_2H_4C_8F_{17})_4$	6396.5040	6396.455	90%
SS7	5'X-AAGAAAGAAGAGA3'	$-C_2H_4C_8F_{17}$	4597.7354	4597.726	98%
SS8	5'X-AAGAAAGAAGAGA3'	$-C_{10}H_{21}$	4291.8956	4291.889	97%
SS9	5'X-AAGAAAGAAGAGA3'	$-(PO_4C_2H_4C_8F_{17})_2$	5277.7012	5277.675	99%
SS10	5'X-AAGAAAGAAGAGA3'	$-(PO_4C_2H_4C_8F_{17})_4$	6637.6327	6637.567	97%
ODN7	5'X-TTCTTTCTTCTCTTTTTTTCTCTTCTTTCTT-X3'	$-C_2H_4C_8F_{17}$	10454.4889	10454.380	99%
ODN9	5'X-TTTCTTCACACTTCTTTCTTCTCTTTTTTTCTCTTC-X3'	$-C_2H_4C_8F_{17}$	11947.7433	11947.640	99%

ODN2

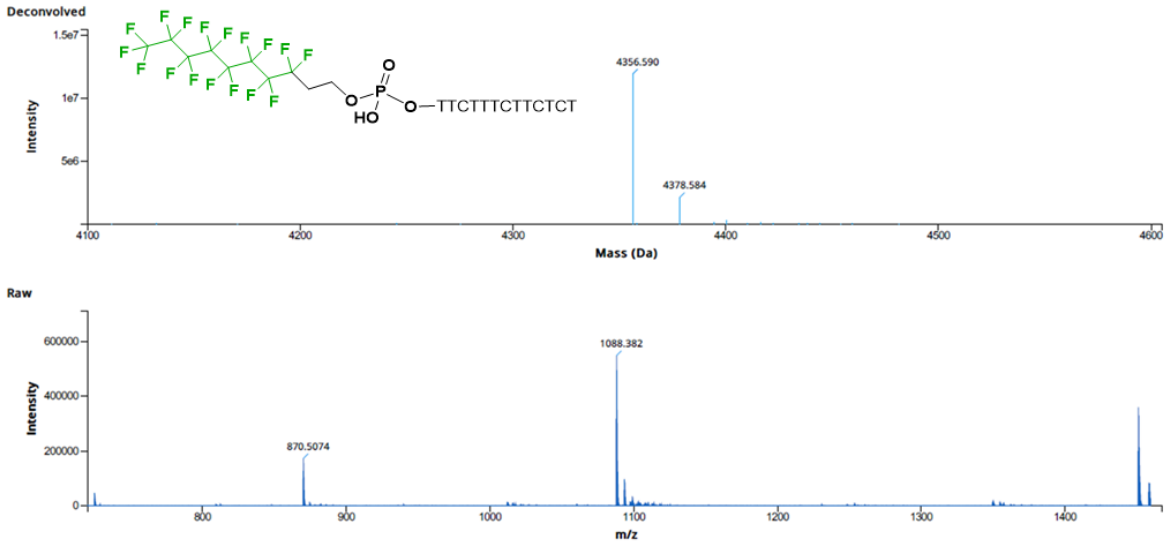

#	Type	Molecule ID	Component	Spectrum	Observed mass (Da)	Expected mass (Da)	Mass error (ppm)	Identity result	Observed TIC RT (mins)
1	Summary	-	-	-	-	-	-	Pass	-
2	Product	Oligo	Oligo	●	4,356.590	4,356.607	-3.7	Pass	2.64

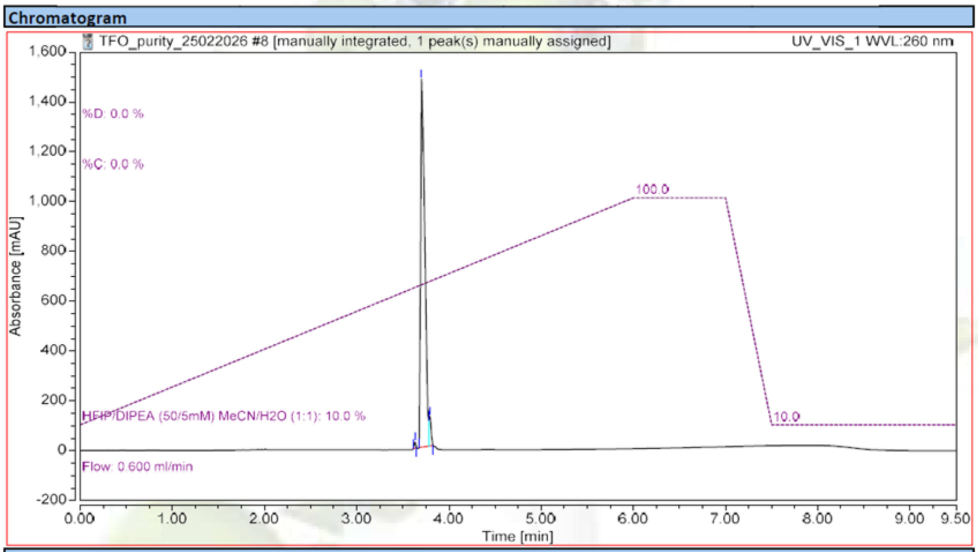

Integration Results

No.	Peak Name	Retention Time min	Area mAU*sec*1000	Height mAU	Relative Area %
1		3.631	24975	24.367	0.52
2		3.703	4636128	1478.460	95.61
3		3.792	187911	119.797	3.88
**Total:**			**4849015**	**1622.624**	**100.00**

Figure S8: Characterisation of ODN2 by RPLC-MS and HPLC analysis (method A).

ODN3

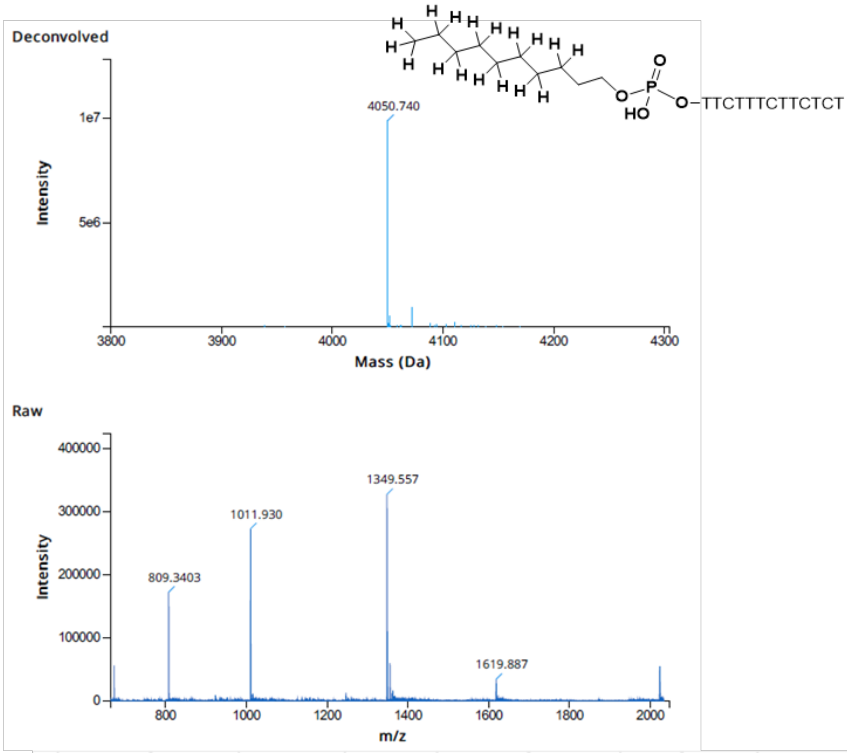

#	Type	Molecule ID	Component	Spectrum	Observed mass (Da)	Expected mass (Da)	Mass error (ppm)	Identity result	Observed TIC RT (mins)
1	Summary	-	-	-	-	-	-	Pass	-
2	Product	Oligo	Oligo	●	4,050.740	4,050.767	-6.5	Pass	2.31

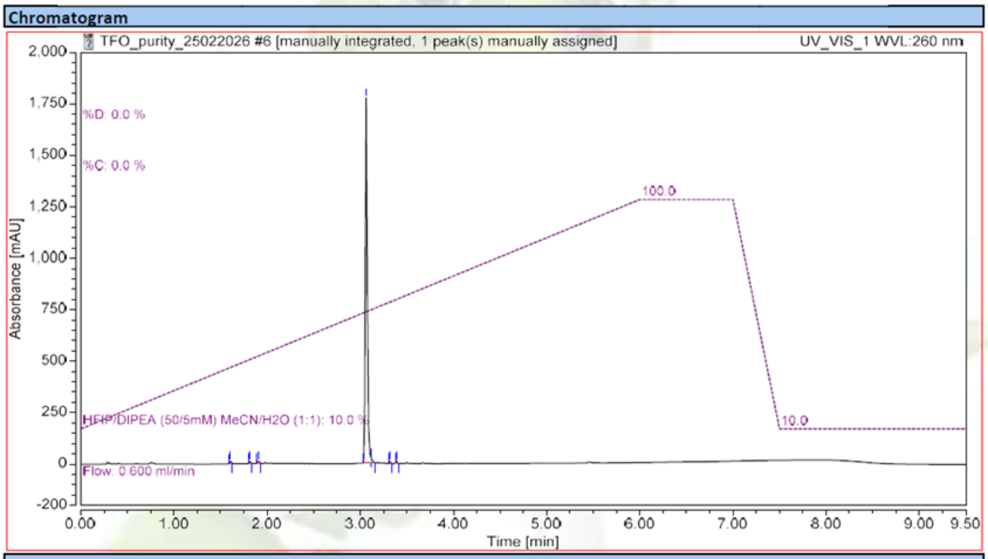

Integration Results

No.	Peak Name	Retention Time min	Area mAU*sec*1000	Height mAU	Relative Area %
1		1.607	14009	12.754	0.50
2		1.814	9040	11.310	0.32
3		1.904	15974	12.557	0.57
4		3.061	2725380	1774.014	97.05
5		3.115	24619	27.741	0.88
6		3.315	10645	11.289	0.38
7		3.388	8556	9.942	0.30
**Total:**			**2808222**	**1859.607**	**100.00**

Figure S9: Characterisation of ODN3 by RPLC-MS and HPLC analysis (method A).

ODN4

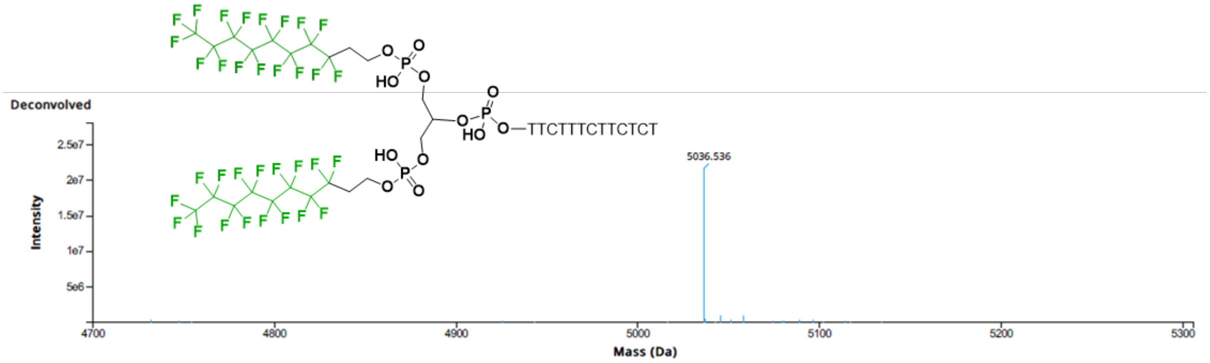

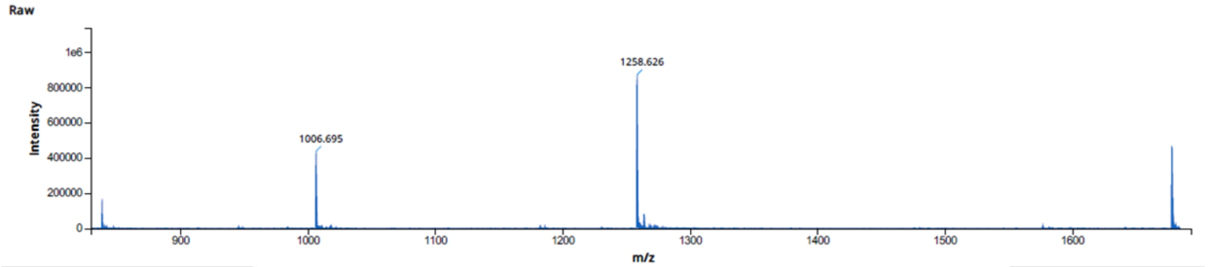

#	Type	Molecule ID	Component	Spectrum	Observed mass (Da)	Expected mass (Da)	Mass error (ppm)	Identity result	Observed TIC RT (mins)
1	Summary	-	-	-	-	-	-	Pass	-
2	Product	Oligo	Oligo All Forms	-	-	-	-	Pass	-
3	Product	Oligo	Oligo n-dT	●	4,732.491	4,732.526	-7.5	Pass	3.23
4	Product	Oligo	Oligo	●	5,036.536	5,036.573	-7.3	Pass	3.23

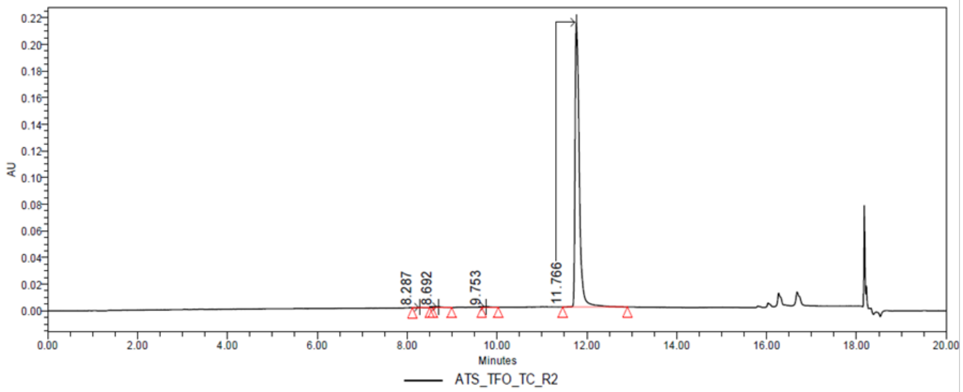

Peak Results

	RT	Area	Height	% Area
1	8.287	2842	358	0.20
2	8.692	5747	617	0.41
3	9.753	2986	423	0.21
4	11.766	1399603	213776	99.18

Figure S10: Characterisation of ODN4 by RPLC-MS and HPLC analysis (method B).

ODN5

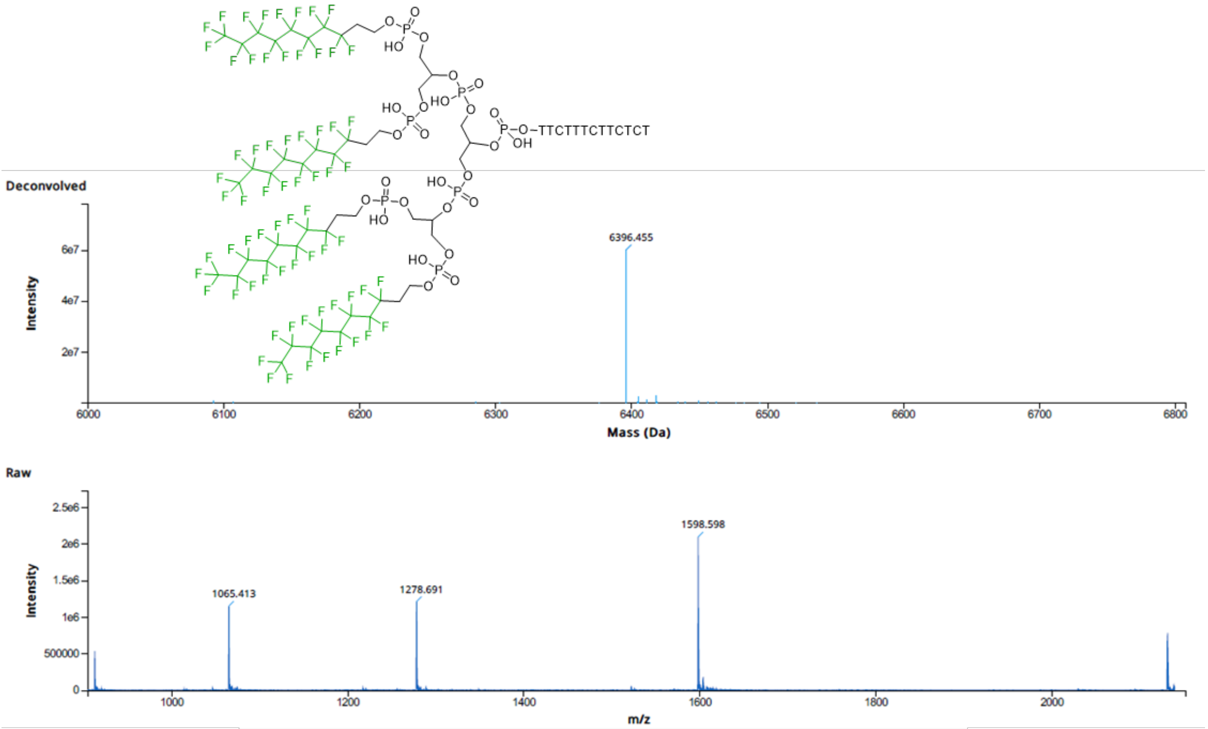

#	Type	Molecule ID	Component	Spectrum	Observed mass (Da)	Expected mass (Da)	Mass error (ppm)	Identity result	Observed TIC RT (mins)
1	Summary	-	-	-	-	-	-	Pass	-
2	Product	Oligo	Oligo All Forms	-	-	-	-	Pass	-
3	Product	Oligo	Oligo	●	6,396.455	6,396.504	-7.6	Pass	3.68

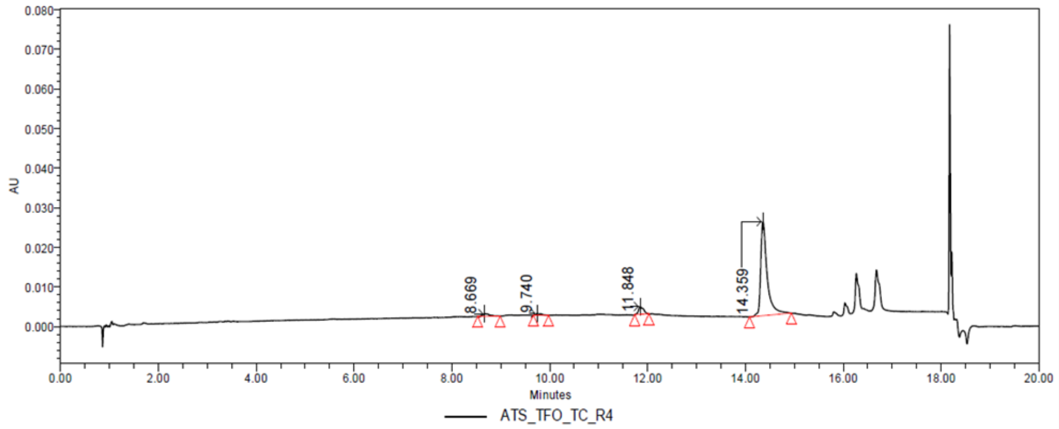

Peak Results

	RT	Area	Height	% Area
1	8.669	6394	658	2.65
2	9.740	3689	469	1.53
3	11.848	13919	1971	5.77
4	14.359	217302	23775	90.05

Figure S11: Characterisation of ODN5 by RPLC-MS and HPLC analysis (method B).

SS7

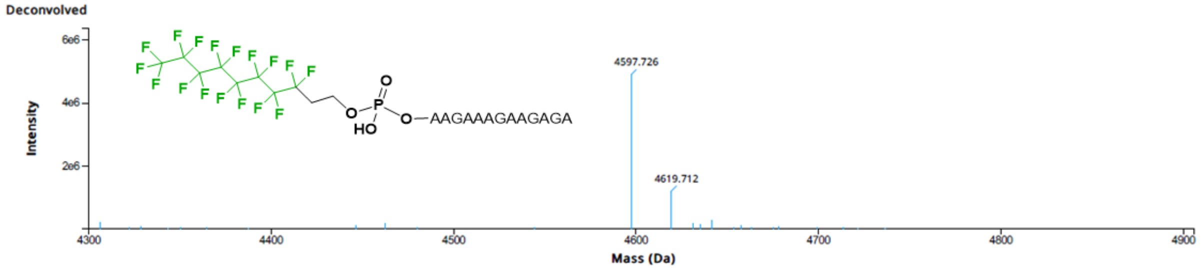

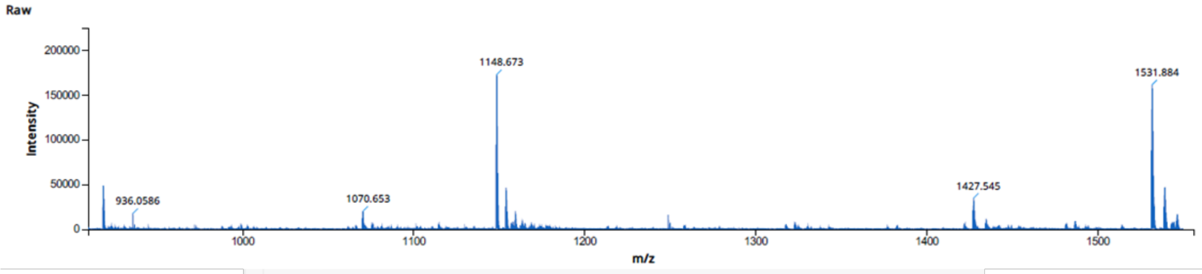

2	Product	Oligo	Oligo All Forms	-	-	-	-	Pass	-
3	Product	Oligo	Oligo -G (gas phase)	●	4,446.673	4,446.686	-3	Pass	2.60
4	Product	Oligo	Oligo -A (gas phase)	●	4,462.674	4,462.681	-1.6	Pass	2.60
5	Product	Oligo	Oligo	●	4,597.726	4,597.735	-2	Pass	2.60

Chromatogram

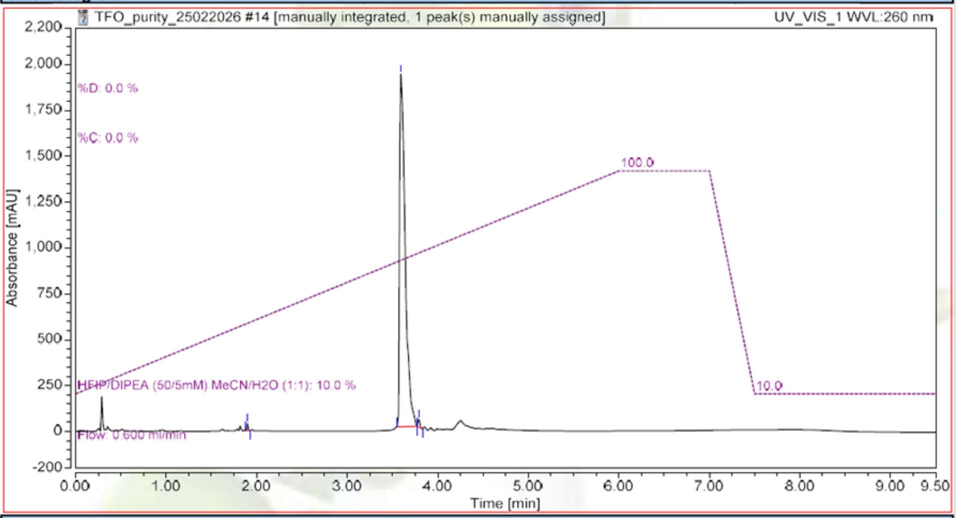

Integration Results

No.	Peak Name	Retention Time min	Area mAU*sec*1000	Height mAU	Relative Area %
1		1.898	38688	41.066	0.47
2		3.592	8134986	1922.061	98.94
3		3.791	48437	41.235	0.59
**Total:**			**8222111**	**2004.362**	**100.00**

Figure S12: Characterisation of SS7 by RPLC-MS and HPLC analysis (method A).

SS8

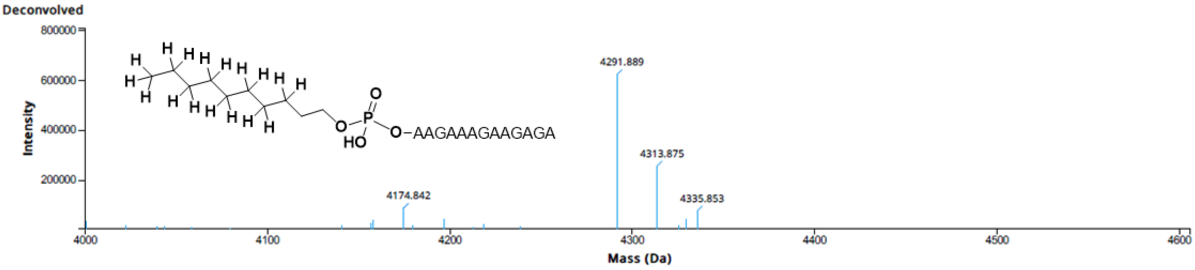

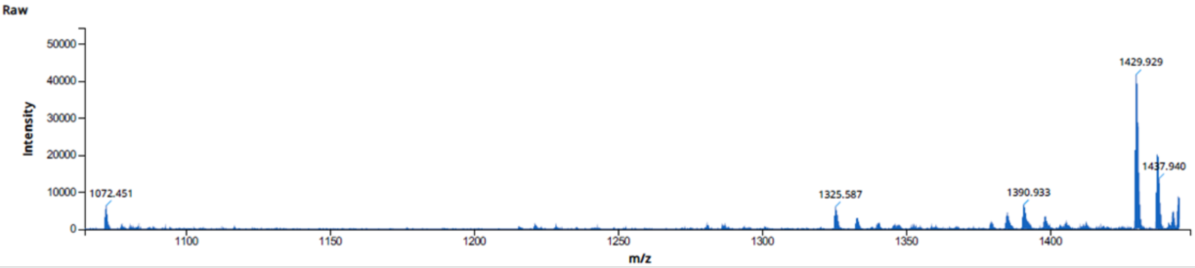

#	Type	Molecule ID	Component	Spectrum	Observed mass (Da)	Expected mass (Da)	Mass error (ppm)	Identity result	Observed TIC RT (mins)
1	Summary	-	-	-	-	-	-	Pass	-
2	Product	Oligo	Oligo All Forms	-	-	-	-	Pass	-
3	Product	Oligo	Oligo -G (gas phase)	●	4,140.816	4,140.846	-7.2	Pass	2.25
4	Product	Oligo	Oligo -A (gas phase)	●	4,156.791	4,156.841	-12.1	Pass	2.25
5	Product	Oligo	Oligo	●	4,291.889	4,291.896	-1.6	Pass	2.25

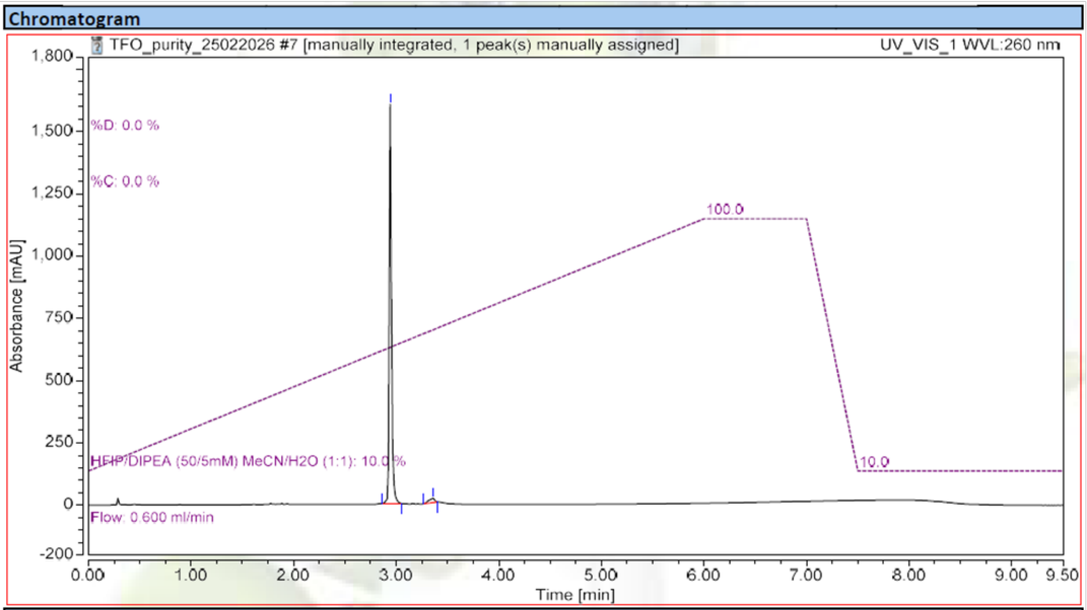

Integration Results

No.	Peak Name	Retention Time min	Area mAU*sec*1000	Height mAU	Relative Area %
1		2.942	2397618	1603.649	97.35
2		3.359	65312	17.869	2.65
**Total:**			**2462931**	**1621.517**	**100.00**

Figure S13: Characterisation of SS8 by RPLC-MS and HPLC analysis (method A).

SS9

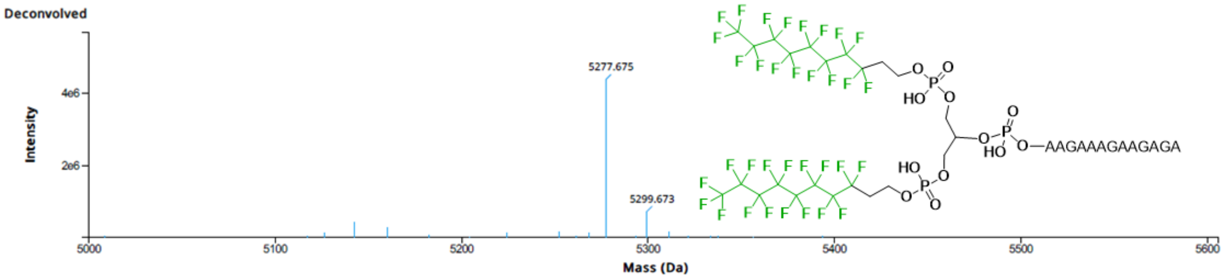

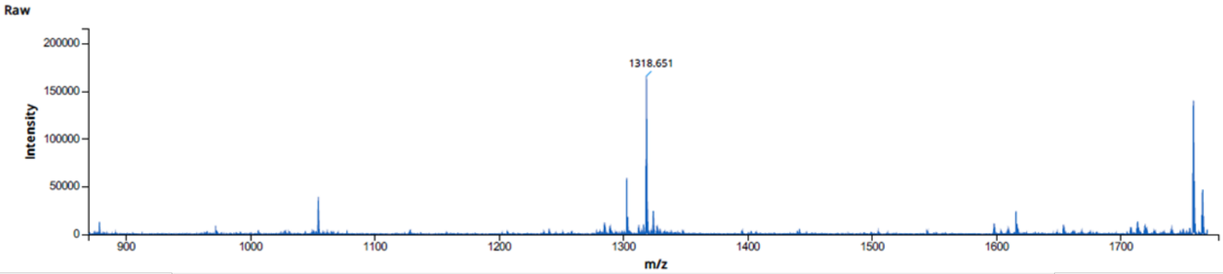

#	Type	Molecule ID	Component	Spectrum	Observed mass (Da)	Expected mass (Da)	Mass error (ppm)	Identity result	Observed TIC RT (mins)
1	Summary	-	-	-	-	-	-	Pass	-
2	Product	Oligo	Oligo All Forms	-	-	-	-	Pass	-
3	Product	Oligo	Oligo -G (gas phase)	●	5,126.618	5,126.652	-6.5	Pass	3.24
4	Product	Oligo	Oligo -A (gas phase)	●	5,142.616	5,142.647	-6	Pass	3.24
5	Product	Oligo	Oligo	●	5,277.675	5,277.701	-5	Pass	3.24

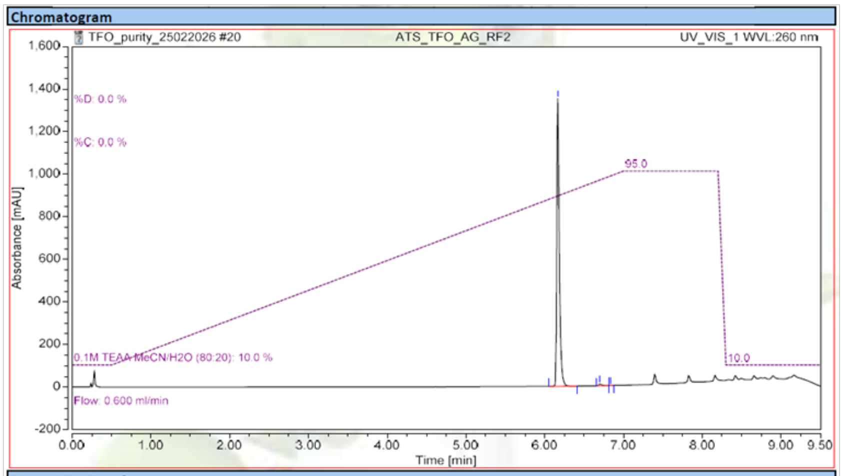

Integration Results

No.	Peak Name	Retention Time min	Area mAU*sec*1000	Height mAU	Relative Area %
1		6.165	2956550	1352.500	99.20
2		6.700	22634	9.721	0.76
3		6.839	1252	0.616	0.04
Total:			2980436	1362.838	100.00

Figure S14: Characterisation of SS9 by RPLC-MS and HPLC analysis (method C). Small peaks after 7 min are present in the blank.

SS10

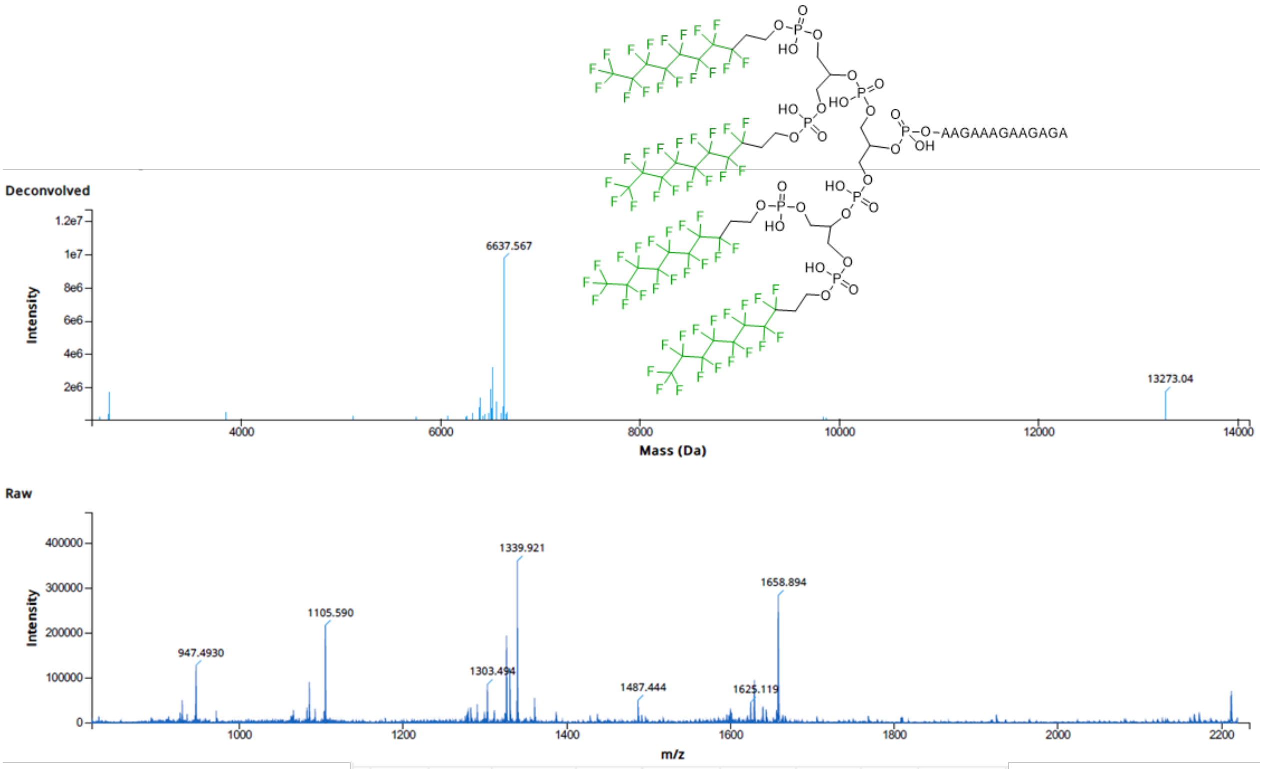

#	Type	Molecule ID	Component	Spectrum	Observed mass (Da)	Expected mass (Da)	Mass error (ppm)	Identity result	Observed TIC RT (mins)
1	Summary	-	-	-	-	-	-	Pass	-
2	Product	6637.6327	6637.6327 All Forms	-	-	-	-	Pass	-
3	Product	6637.6327	6637.6327 n-dA	●	6,324.507	6,324.575	-10.8	Pass	3.69
4	Product	6637.6327	6637.6327 -G (gas phase)	●	6,486.537	6,486.583	-7.1	Pass	3.69
5	Product	6637.6327	6637.6327 -A (gas phase)	●	6,502.510	6,502.578	-10.4	Pass	3.69
6	Product	6637.6327	6637.6327	●	6,637.567	6,637.633	-9.9	Pass	3.69

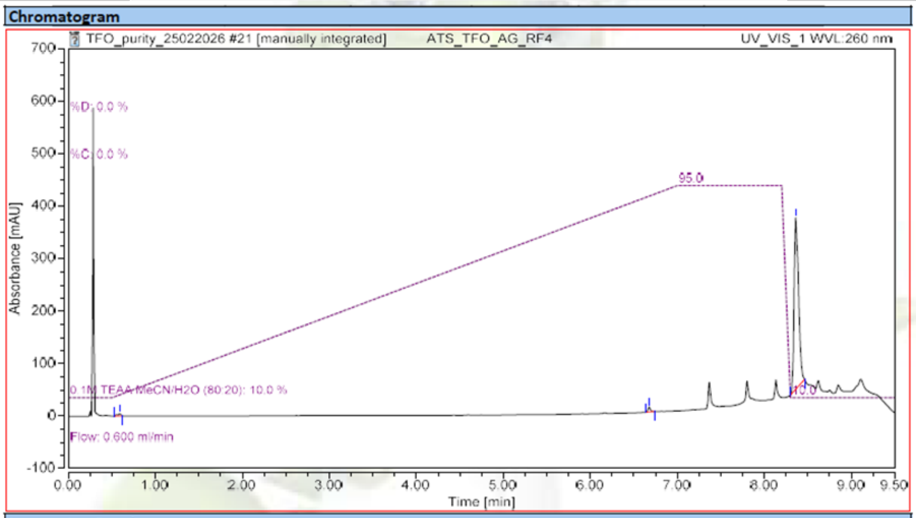

Integration Results

No.	Peak Name	Retention Time min	Area mAU*sec*1000	Height mAU	Relative Area %
1		0.591	12805	5.225	1.08
2		6.675	17854	9.482	1.51
3		8.360	1151692	327.377	97.41
**Total:**			**1182352**	**342.083**	**100.00**

Figure S15: Characterisation of SS10 by RPLC-MS and HPLC analysis (method C). Small peaks after 7 min are present in the blank.

ODN7

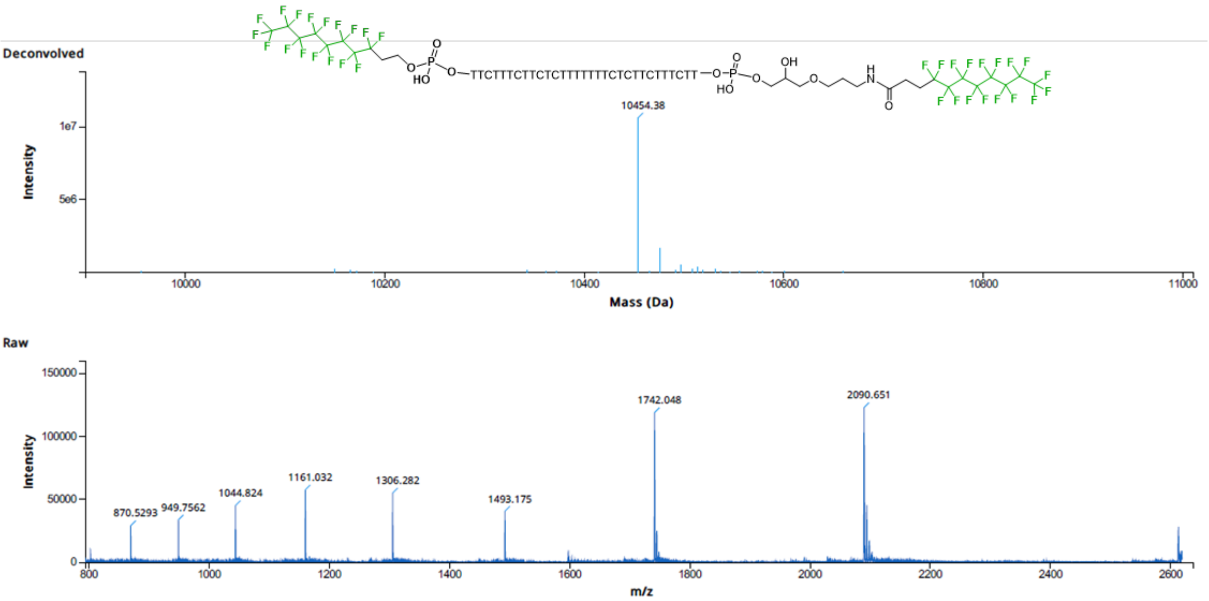

#	Type	Molecule ID	Component	Spectrum	Observed mass (Da)	Expected mass (Da)	Mass error (ppm)	Identity result	Observed TIC RT (mins)
1	Summary	-	-	-	-	-	-	Pass	-
2	Product	Oligo	Oligo All Forms	-	-	-	-	Pass	-
3	Product	Oligo	Oligo n-dT	●	10,150.35	10,150.44	-9.6	Pass	2.91
4	Product	Oligo	Oligo n-dC	●	10,165.33	10,165.44	-11.1	Pass	2.91
5	Product	Oligo	Oligo	●	10,454.38	10,454.49	-10.3	Pass	2.91

Chromatogram

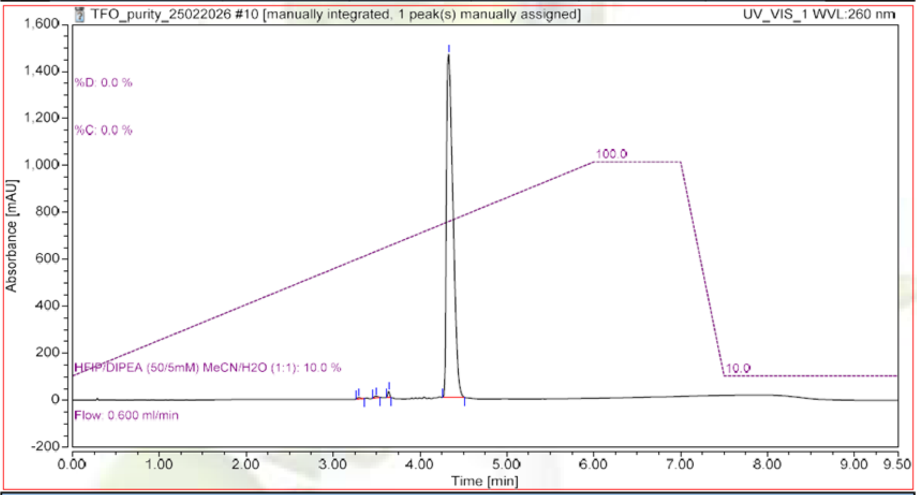

Integration Results

No.	Peak Name	Retention Time min	Area mAU*sec*1000	Height mAU	Relative Area %
1		3.294	17168	6.265	0.22
2		3.496	23826	7.679	0.31
3		3.640	34385	25.122	0.44
4		4.329	7655826	1461.623	99.03
**Total:**			**7731206**	**1500.689**	**100.00**

Figure S16: Characterisation of ODN7 by RPLC-MS and HPLC analysis (method A).

ODN9

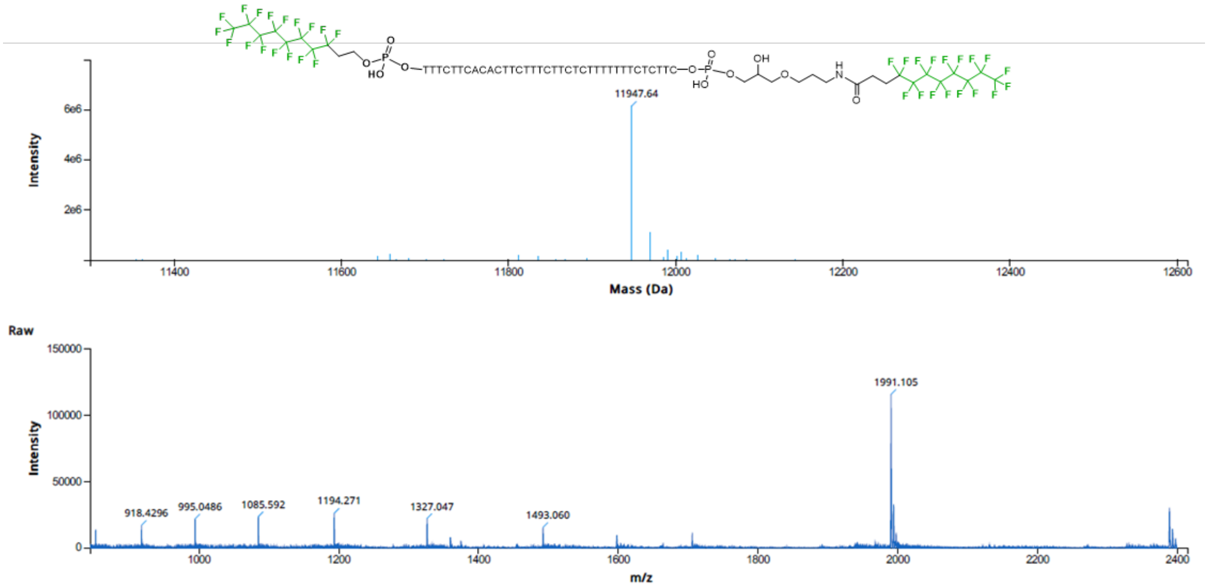

#	Type	Molecule ID	Component	Spectrum	Observed mass (Da)	Expected mass (Da)	Mass error (ppm)	Identity result	Observed TIC RT (mins)
1	Summary	-	-	-	-	-	-	Pass	-
2	Product	Oligo	Oligo All Forms	-	-	-	-	Pass	-
3	Product	Oligo	Oligo n-dT	▲	11,643.60	11,643.70	-8.2	Pass	2.87
4	Product	Oligo	Oligo n-dC	▲	11,658.59	11,658.70	-8.9	Pass	2.87
5	Product	Oligo	Oligo -A (gas phase)	▲	11,812.59	11,812.69	-8.1	Pass	2.87
6	Product	Oligo	Oligo	▲	11,947.64	11,947.74	-8.9	Pass	2.87

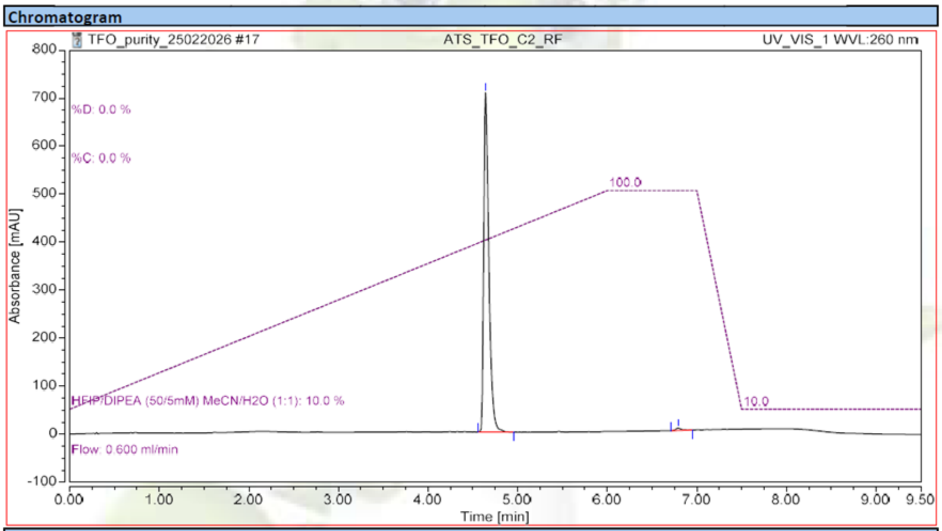

Integration Results

No.	Peak Name	Retention Time min	Area mAU*sec*1000	Height mAU	Relative Area %
1		4.642	2828212	707.638	99.21
2		6.789	22609	5.006	0.79
**Total:**			**2850821**	**712.644**	**100.00**

Figure S17: Characterisation of ODN9 by RPLC-MS and HPLC analysis (method A).

References

1 C. Adam, L. Yang, D. S. L. Cockroft, *Angewandte Chemie*, 2015, **54**, 1164–1167.
